# Residual Stresses and Micro‐voids Propel Metal Diffusion for Filament‐Based Memristors

**DOI:** 10.1002/advs.202416305

**Published:** 2025-05-09

**Authors:** Joel Y.Y. Loh, Jason Yuan, Samuel Moor‐Smith, Adam Glustein, Gloria Vytas, Will Taylor, Andres Lombo, Richard J. Curry, Nazir Pyarali Kherani

**Affiliations:** ^1^ Department of Electrical and Computer Engineering University of Toronto Toronto Ontario M5S 3G4 Canada; ^2^ Department of Electrical and Electronic Engineering Photon Science Institute University of Manchester Alan Turing Building Oxford Rd Manchester M13 9PY United Kingdom; ^3^ Department of Physics University of Toronto Toronto Ontario M5S 3G4 Canada; ^4^ Department of Materials Science and Engineering University of Toronto Toronto Ontario M5S 3E4 Canada

**Keywords:** memristor, sputtering, stress, thin film

## Abstract

Metal filamentation based mechanisms have the advantage of a high switching current ratio, yet typically require high switching voltages to activate the memristive device due to the primary mechanism of atomic vacancy filling and movement. Herein, Introducing non‐reactive nitrogen gas during plasma sputtering of silver is shown to prime the overlying metal nitride layer to achieve low threshold switching at applied biases of below 60 millivolts. Residual nitrogen species within the silver under‐layer promote the creation of nano‐sized void defects within the superjacent dielectric layer, which, coupled with residual stresses in the gigapascal range, enable sub‐micron filamentation growth. These memristor devices function similarly to potassium ion channels, displaying current growth and relaxation patterns that align with the Hodgkin‐Huxley model, and as such are amenable to the development of artificial neuron structures. Further, a diverse set of neuromorphic behaviors not seen within typical metal filamentation based memristors is observed. This includes multi‐peak synaptic weight changes in the device's response to spiked stimuli. Both the switching voltages and neuromorphic properties are linked to the nitrogen‐argon pressure during silver deposition. Interestingly, these devices also exhibit lateral growth of silver filamentation across the surface of the metal nitride thin film layer with gaps of more than a hundred micrometers, suggesting that the underlying silver undergoes accumulation and breakthrough. The filling of large micro‐voids with Ag generates large nanoparticles that easily propagate, enabling a large diffusion front and faster filamentation time, whereas small micro‐voids create a bottleneck in the filamentation process. Additionally, the introduction of residual stresses in conventional diffusion theory indicates greater dendritic interconnectivity and thus electrode to electrode connection. This study demonstrates that the facile incorporation of non‐reactive gases during the sputter‐deposition of a metal electrode opens a path to unique material mechanisms that facilitate the development of versatile memristors.

## Graphical abstract

Introducing non‐reactive nitrogen during silver sputtering enables low‐voltage (<60 mV) switching in memristors via nano‐void formation and residual stresses in the gigapascal range. This promotes sub‐micron filamentation and neuromorphic behaviors, including multi‐peak synaptic responses. Nitrogen‐argon pressure influences switching and lateral filament growth. Residual stress enhances dendritic interconnectivity, accelerating Ag diffusion and electrode bridging in these neuromorphic devices.

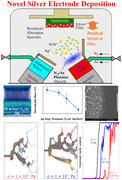

## 1 Introduction

Conductive filament memristors–a prominent category among memristors–are of significant interest in the field of neuromorphic computing. The underlying mechanism in these memristors is filamentation, a phenomenon where conductive strands either form or rupture, directly affecting the device's electrical resistance. Recent advancements in the field have shed light on the role of various types of defects that influence the filamentation mechanism. These defects, which include vacancies,^[^ ^1^, ^2^, ^3^, ^4^, ^5^ ^]^ interstitials^[^ ^6^, ^7^, ^8^, ^9^ ^]^ substitutional^[^ ^10^, ^11^, ^12^, ^13^ ^]^ or anti‐site defects^[^ ^14^, ^15^, ^16^ ^]^ extrinsic point defects, metal cation defects, and extended defects such as dislocation networks and grain boundaries, can modulate the device conduction processes and thus influence the filamentation mechanism. Understanding and tailoring these defects can be a promising step toward memristors with controlled conduction pathways, potentially improving device performance and reliability. For example, a low reactive sputtering pressure during the fabrication of ZnO‐based memristors leads to non‐volatile memory behavior due to high oxygen vacancy concentrations and a large volume of grain boundaries, enabling favorable dendritic filamentation.^[^ ^17^ ^]^ The use of higher sputtering pressures, lower oxygen vacancy concentrations, and directional film growth confines filamentation to columnar grain structures such that filament rupturing occurs when oxygen displaces the filament sites. It is clear that fabrication processes influence the type and concentration of defects and in turn, underpin control over the realized memristive behaviors.

Crucial initial steps in the filament growth process include the ionization of the active electrode material into cations under the influence of an applied electric field, the transport of these cations toward the inert electrode across the dielectric thin film under high field, and the reduction of these cations to atoms which leads to the nucleation and growth of metal clusters and ultimately the metallic nanofilaments. The efficiency of this process can be influenced by several factors, including the metal work function, cation mobility, electric field strength, temperature or Joule heating effects, charge environment, interfacial defects between electrodes and switching materials, and device structure. Of these factors, the role of micro‐void defects, which are substantially larger than atomic‐scale defects, and residual stress in non‐ferroelectric materials are not well examined in the literature. Residual stresses can deform lattice arrangement and create more space for ions to move, thus increasing cation mobility.

In non‐epitaxial depositions such as sputter deposition, atomic clusters that underlie island formation lead to compressive film stress due to their high surface energy. When the islands expand, leading to island‐to‐island contact, the closing of the gaps between islands transacts the interfacial energy associated with cracks and grain boundaries for higher surface energy. This results in compensating tensile stress within the film. With increasing densification and thickness, compressive forces arise due to energetic bombardment by neutral Ar and sputtered ions. If the energetic bombardment is hindered by a high pressure in the plasma sheath, the compressive forces can be negated resulting in net tensile stress. In particular, for AlN films, their residual stresses under various deposition parameters have been well studied.^[^ ^18^, ^19^, ^20^ ^]^ The growth of AlN films using reactive nitrogen sputtering of aluminum^[^ ^21^ ^]^ shows that high sputtering pressures induce loosely packed (101) planes and reduce the formation of closed‐packed (002) planes. At higher pressures, the films exhibit tensile stress due to atomic self‐shadowing (bombardment at an oblique angle) and gas scattering (loss of kinetic energy), which also contributes to low densification during film growth. Thus, AlN films are shown to be able to accommodate both compressive and tensile residual stresses depending on growth conditions.

In this work, we show that control over both micro‐voids and residual stresses in the switching medium, achieved by virtue of the deposition approach of the underlying silver electrode, can induce silver diffusion into the switching medium even before applying an external voltage. The presence of these voids and residual stresses enables variation of memristive behavior at low switching voltages. We also show that dendrite formation and growth behavior significantly differ in the presence of stress, resulting in visibly observable filamentation growth over a long distance of 100 µm at 1 V bias. This deposition approach is unusual in that it does not involve directly altering the deposition of the switching medium which is nominally Aluminum Nitride^[^ ^22^, ^23^, ^24^, ^25^, ^26^, ^27^ ^]^ but by simply introducing nitrogen (N_2_) to the Argon (Ar) plasma sheath during the deposition of the silver electrode (the first layer of the device). In this work, only the N_2_‐Ar sputtering pressure during silver deposition was varied while the sputtering pressure of the aluminum nitride deposition was unchanged. Nitrogen does not react with the silver but can be trapped within the silver film, becoming a residual gas that interacts with the subsequent deposition of the overlying aluminum nitride. This changes the compositional profile and binding energy profile of the layer stack. The lattice strain^[^ ^24^ ^]^ introduces net tensile residual stress with a reduction in local stress that decreases film hardness without inducing exfoliation of the bi‐layers, while adding micro‐voids to the aluminum nitride film, whereby silver diffusion can occur spontaneously. We thus investigate the effects of N_2_‐Ar plasma induced silver deposition at varying pressures, and in vertical, diagonal, and lateral memristor device configurations to understand how these novel types of defects and environment control memristive behavior.

## 2 Results and Discussion

**Figure 1:**
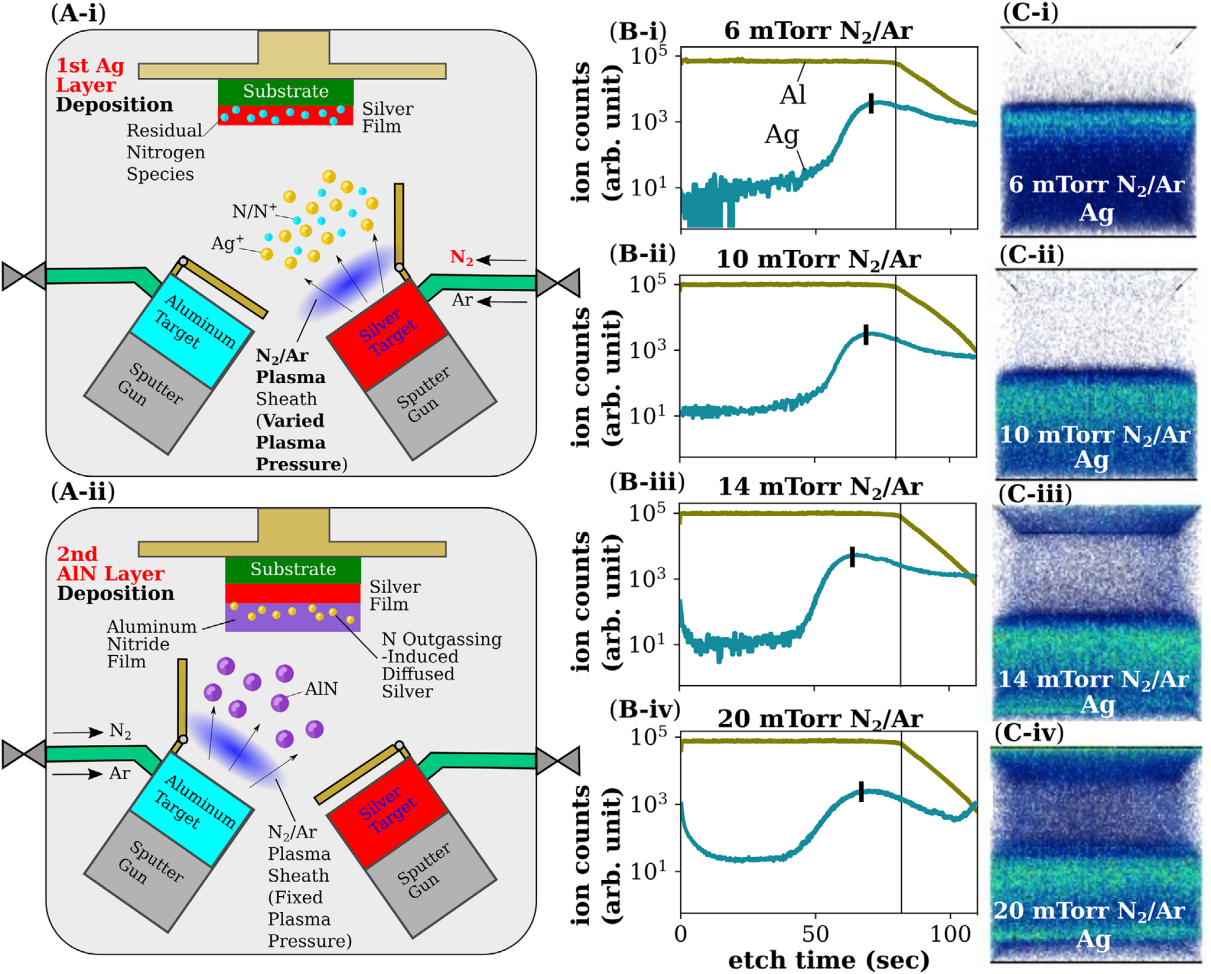
Schematic of deposition approach targeting the underlying silver layer whereby its diffusion properties are varied with increasing N_2_‐Ar pressures. A‐i) A schematic of the vacuum sputter‐deposition chamber where the silver target is bombarded by a N_2_‐Ar plasma mixture. The plasma sheath density can be controlled by the plasma pressure. This introduces residual nitrogen species in the silver layer. A‐ii) The subsequent deposition of aluminum nitride (AlN) by reactive sputtering of Al metal target with a N_2_‐Ar plasma mixture. The AlN deposition proceeds under a low N_2_‐Ar pressure for all devices and samples. B) The time of flight‐secondary ion mass spectroscopy (TOF‐SIMS) of the AlN/Ag bilayer films prepared with Ag depositions 6–20 mTorr N_2_‐Ar pressures. TOF‐SIMS is a sensitive surface analysis method that uses a primary ion beam to eject secondary ions from a sample for high‐resolution compositional analysis and concentration estimation through depth profiling and mass spectrometry. The Ag profile extends deeper beyond the AlN interface (denoted with a black line) with increasing pressures i‐iii). At 20 mTorr iv), the silver profile at the interface is similar to the 6 mTorr sample but shows higher silver concentration in the AlN bulk to the AlN surface. All ion counts are shown on a log scale. The Ag peak and Al decay at the interface are measurement artifacts. C) The TOF‐SIMs 3D rendering of the Ag concentration profiles in the AlN/Ag film under varying N_2_‐Ar pressures. The blue‐teal pixels are indicators of Ag ion detection. The bottom layer is the underlying silver film.

The addition of N_2_ to the Argon plasma mixture during sputter deposition of the underlying silver (Ag) film induces silver diffusion into the overlaying Aluminum Nitride (AlN) layer during its deposition (**Figure** **1**A‐i). Typically, N_2_ is not commonly introduced during non‐reactive sputtering of metals, as the process of dissociating N_2_ results in a complicated mixture of neutral N atoms, N_2_ molecules, N_2_^+^ and N^+^ ions, and excited N* and N_2_* species within the plasma sheath. By contrast, Argon is commonly used due to its chemical inertness,^[^ ^28^ ^]^ resulting in a plasma sheath consisting of Ar^+^ ions and Ar neutrals. Under a mixed N_2_‐Ar plasma discharge sputtering of Ag and the essentially non‐reactive nature of Ag considering the high enthalpy of formation of silver nitride, the growth of the Ag film is thus likely to include residual N and N_2_ molecules which can subsequently diffuse during the deposition of the AlN layer or outgas upon unloading the Ag film from the sputter‐deposition chamber. For all devices, deposition of the AlN layer was carried out by reactive sputtering of a metallic Al target and N_2_‐Ar mixture (Figure 1A‐ii). All AlN layers are deposited using N_2_:Ar flow ratio of 8:12 sccm and a conventionally low sputter‐deposition (plasma) pressure of 2.5–3.0 mTorr. All sputtering pressures, unless indicated otherwise, correspond to that used during the underlying Ag layer deposition.

We found that varying the N_2_:Ar flow ratio for the Ag deposition does not significantly change the Ag diffusion concentration profile, however, small changes in the sputter‐deposition pressure results in significant tailing of the Ag diffusion into the AlN layer. The TOF‐SIMs profiles show (Figure 1B) that at 6 mTorr N_2_‐Ar pressure (Figure 1B‐i) the Ag profile already exhibits trace silver beyond the Al/Ag interface (which is presumed to be where the Al signal begins to decay) with negligible Ag concentration (below an ion count of 10) in the top 1/3 of the AlN layer thickness. By contrast for an AlN/Ag bilayer without N_2_ during Ag deposition (Figure S1, Supporting Information), the Ag at the interfacial region has a sharper peak and a steep decrease in Ag to a negligible concentration for more than half of the AlN layer. The Ag peak broadening effect becomes more pronounced in samples with greater N_2_‐Ar plasma pressure, and the position of the Ag peak extends ahead of the interfacial region as seen in samples deposited at 10 and 14 mTorr pressures (Figure 1B‐ii, iii). This indicates that there is only trace Ag above the interface (and it is noteworthy that Ag is fairly ionizable for TOF‐SIMs detection). With a 20 mTorr N_2_‐Ar plasma pressure associated bilayer, the Ag interface peak does not extend further, but the Ag profile is noticeably broadened compared to the other samples. The Ag concentration profile at the interfacial region in the AlN layer of all samples thus seems to follow a quasi‐log profile where the logistic growth rate slows down with increasing Ag pressure as reflected by the broadening of the peak. This suggests that the Ag diffusion profile is dependent on an initially low availability of Ag atoms for diffusion. It will be shown later that the presence of micro‐voids above the interface of the AlN/Ag bilayer is partially filled with Ag nanoparticles, which would indicate that the initial source of Ag diffusion is associated with these nanoparticles just above the interface and not the Ag layer. Indeed, a cross‐sectional SEM‐BSE examination of an 14 mTorr N_2_‐Ar bilayer shows Ag nanoparticles of less than 10 nm diameter and just above the interface (Figure S2, Supporting Information). The 20 mTorr sample also showed a significantly greater concentration of trace Ag in most of the AlN layer. The Ag concentration at the sub‐surface of the AlN layer is high. An examination of the surface of the AlN layer by SEM and TOFSIMS surface profiling shows the presence of blisters where circular particles of up to 1 µm diameter can be seen (Figure S3a, Supporting Information). We also deposited AlN first before depositing Ag with N_2_/Ar (Figure S3b, Supporting Information), where Ag was also detected in high concentrations in the underlying AlN layer, indicating the dual directionality of the diffusion mechanism. In all, this unconventional plasma gas mixture of N_2_‐Ar based unreactive sputter deposition of Ag can thus induce extensive Ag diffusion at varying concentration profiles.

**Figure 2:**
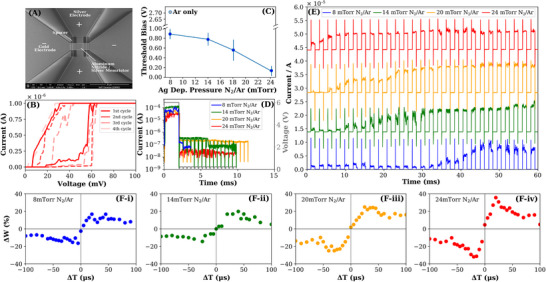
Electronic behavior of AlN/Ag vertically configured memristors under various deposition N_2_‐Ar pressures of the underlying Ag electrode. A) SEM image of the memristor device. The bottom positive Ag electrode is capped with thicker AlN spacer layers at the electrode edges. The top negative gold electrode is deposited, with conventional Ar sputtering, over the spacer layers, and over the AlN/Ag bilayer. The scale bar is 30 µm. B) The hysteresis IV plot for the device associated with 24 mTorr pressure deposition. Over four cycles, the switching threshold is 50–60 mV, while the return loop has a switching threshold of 20–40 mV. C) The switching thresholds of sets of 10 devices each over a range of Ag deposition pressures. A device made with only Ar plasma during Ag deposition is shown to have a threshold voltage of ≈2.72V. The threshold bias decreases from 850 ± 100 V to 50 ± 20 mV. D) The relaxation behavior after an initial 10 V square pulse of 2.5 ms duration. The device of 14 mTorr relaxed after a fairly long period of 4 ms. E) The current growth behavior over a sequence of square 2 V pulses. Devices associated with higher Ag deposition pressures had rapid growth to current saturation. F) The STDP plots of devices fabricated at various Ag deposition pressures. Higher Ag deposition pressures display greater conductivity changes when subject to spiked stimuli.

The memristive behavior is significantly aided by this Ag sputtering approach and is further controllable by the plasma pressure during the Ag deposition. The memristor device (**Figure** **2**a) with gold overlaying electrode and with the underlying Ag layer extended laterally as an electrode, is fabricated at different N_2_‐Ar plasma pressures for the Ag deposition while holding all other device and sputtering parameters constant. We found that with the introduction of N_2_ in Ar at 8 mTorr substantially decreases the switching voltage of the 50 nm AlN layer memristor from several volts to less than one volt. With increasing N_2_‐Ar pressures the switching voltages decrease down to 60 mV using 24 mTorr deposition pressure (Figure 2C). The switching voltage decreases somewhat with increasing cycles, while the reversal switching voltage doubles from 20 to 40 mV (Figure 2B). The current relaxation time of a single 10 V pulse also extends from 0.2 ms to 1 ms with increasing pressure and has the shortest relaxation time of 0.2 ms in the 8 mTorr device, while the 14, 20, and 24 mTorr devices have relaxation times of 0.3, 0.9, and 1 ms respectively (Figure 2D). The current growth behavior (Figure 2E) with 2 V pulse train also becomes more sensitive to pressure‐ exhibiting a transition from slow current growth for the 8 mTorr device to a sharp rise within 4 pulses for the 24 mTorr device. This indicates that filament interconnectivity occurs more rapidly before saturating at the electrode, implying a high Ag nanoparticle propagation or an additional potential driving greater interconnectivity. Faster interconnectivity of the Ag filament will connect the electrodes more rapidly allowing the device to achieve conductivity.

We report the results of the synaptic weight change in AlN/Ag memristors under STDP (Spike‐timing‐dependent plasticity) testing at various device formation pressures. Figure 2‐F illustrates that increasing formation pressure expands the range of synaptic weight changes—the difference between the max and min *Δw*—that the memristor exhibits. This suggests that there is an increasing degree of Ag mobility due to an alteration in its electrochemical behavior by the surrounding environment.^[^ ^29^ ^]^ Figure 2‐F‐i and Figure 2‐F‐iv indicate that the max magnitude of the synaptic weight change at 24 mTorr is about double that of the 8 mTorr devices when triggered with a similar spiked stimulus—the device conductivity changes twice as much when stimulated with the same spiked input. Another observation of importance is that the maximum synaptic weight change of the 8 mTorr device is similar to that of the 14 mTorr devices. Concurrently, the maximum weight change of the 20 mTorr device is similar to that of the 24 mTorr device. However, the two higher pressure devices differ greatly from those at the two lower pressures which suggests a threshold pressure that governs when a significant conductivity change may be observed. This threshold pressure directly affects the mobility of Ag^+^ ions and the memristor's response to temporally correlated stimuli. At pressures lower than the threshold, Ag^+^ ions are less mobile and therefore unable to traverse the medium easily to build a conductive filament. When below this threshold pressure, the AlN structure seems only to be partially altered by out‐gassing effects and is insufficient to trigger faster filamentation—this explains the similarities between Figure 2‐F‐i and Figure 2‐F‐ii. Once the pressure has exceeded the minimum threshold, the residual stresses induced in the AlN enable increased Ag^+^ ion mobility, allowing for rapid filamentation. Faster filamentation explains the higher device conductivity and quicker device response observed in Figure 2‐F‐iii and Figure 2‐F‐iv. For temporal differences outside of the region where spikes occur, the higher‐pressure devices plateau and approach 0% weight change at a slower rate than those at lower pressures. Moreover, the results indicate that the Ag/AlN memristors can achieve a conductive state on a microsecond scale. The microsecond timescale is a significant improvement over the millisecond timescale of action potentials in biological neurons and implies faster “learning” processes.^[^ ^30^, ^31^ ^]^ Therefore, we summarize the two notable findings of the Ag/AlN memristor in the vertical configuration. The first takeaway is that the magnitude and range of synaptic weight changes are impacted by the Ag sputtering N_2_‐Ar pressure; increasing pressures result in a larger synaptic weight change only once a minimum pressure threshold is exceeded. The second takeaway is that the vertical Ag/AlN memristors are capable of exhibiting STDP learning behavior on a microsecond scale—a significant speedup to the millisecond timescale of action potentials in biological neurons. Given these findings, memristors deposited with N_2_‐Ar during metal deposition can have excellent potential for implementing spiked neural networks (SNNs) for neuromorphic computing applications.

We now compare our AlN/Ag memristors with biological neurons. The Hodgkin‐Huxley (HH) model^[^ ^32^ ^]^ is a set of equations that describe the electrical characteristics of biological neurons Electrical stimuli are conducted through the neuron's transmission lines due to the movement of ions in and out of the neuron membrane. This process is referred to as an action potential. The permeability of the neuron to ions at different points in the action potential is governed by the potential difference of the neuronal membrane. Hence, the conductivity of the neuron can be said to depend on neuron permeability. The capacity of neurons to have varying conductivity is described as memristive and modeled as such.^[^ ^33^ ^]^ In the HH model, gating parameters dependent on the membrane potential mimic the neuron permeability to ions. The gating parameter *n* is a time and membrane potential‐dependent variable that represents the probability K^+^ ion channels are open and allows K^+^ ions to move across the neuron membrane. The gating parameters *m* and *h* represent the probability that the Na^+^ ion channels are open and permeable to Na^+^ ions, and closed or impermeable to Na^+^ ions, respectively. Similarly, these parameters are dependent on time and membrane potential. Simulations of voltage‐clamped neurons approximate the response of the Ag/AlN memristor. In the Ag/AlN memristor, the gating parameter *n* can analogously model the permeability of the AlN dielectric to Ag^+^ ions when a switching pulse is applied. The fitting of current relaxation behavior associated with 14 mTorr devices (Figure S5, Supporting Information) demonstrated that at rest potential (i.e., the voltage of the memristor when no stimulation is applied) the gating probability was close to zero. In contrast, the gating probability was in the range of 0.7 to 0.9 after the applied switching pulse reached its maximum value indicating that a stimulation exceeding threshold has been applied. Additionally, the memristor conductance trend is maintained by adapting constants to the values of the Ag/AlN memristor in the HH‐model. For instance, the maximum conductance value that the memristor could reach was on the order of 10^−2^ mS cm^−2^ which is on the same magnitude scale as the actual max conductance of AlN. Moreover, the gating probability changes accordingly with the memristor conductivity. In a high‐resistive state, the low current indicates a low permeability of Ag^+^ ions and hence the low gating probability. In contrast, a higher gating probability corresponds to a larger current being carried because of the higher permeability and initial presence of Ag in the AlN dielectric.

**Figure 3:**
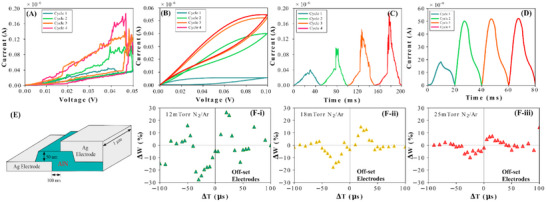
Synaptic characteristics of AlN/Ag memristors in the vertical orientation and STDP response of offset electrodes. A) Current response of a voltage sweep on the AlN/Ag memristor. Each cycle of the voltage sweep sees an increase in the growth of the current indicating increasing conductivity. B) Simulated K^+^ ion current response according to the HH model equation. Each cycle of the voltage sweep also sees a growth in the current similar to a memristor. C) Current response of the AlN/Ag memristor over time subject to four consecutive pulses. Maximum current is observed to increase with each cycle demonstrating that the memristor has not saturated. D) Simulated K^+^ ion current response over time. The K^+^ ion current has the largest growth from the first to the second cycle and reaches its maximum by the fourth cycle. E) Design of the AlN/Ag memristors configured with offset electrodes. F) STDP plots of the AlN/Ag memristor in the offset electrode orientation. At lower pressures, the synaptic weight change shows additional peaking behavior after its maxima and increasing pressure sees the STDP response approach that of vertical orientations. Small secondary peaks are observed in ≈−40 and +40µs in both Figure (F‐i) and (F‐ii).

As further evidence that the HH‐model is valid for our devices, we also examined the similarities in the I‐V characteristics of the memristor and the K^+^ ion channel. Using a 0–500 mV voltage sweep on the AlN/Ag memristor devices, the current‐voltage relationship is illustrated in **Figure** **3**A. A normalized (with respect to pulse duration) growth in the current after each cycle of the voltage sweep is shown in Figure 3C —normalization adjusts the pulse duration in each cycle to be equal. In Figure 3B and Figure 3D, we show the simulated current‐voltage relationship obtained by applying a 10 mV triangle voltage waveform at 100 Hz to an ideal K^+^ ion channel as well as the current growth over time after the application of each cycle. Parameters for the ideal K^+^ channel used the max conductance value of 36 mS cm^−2^ and a potassium rest membrane potential of 12 mV.^[^ ^32^ ^]^ The same triangle waveform stimulus is used in simulations of the Na^+^ and combined ion model.

Our simulations of the K^+^, Na^+^, and hybrid ion current according to the HH‐model find that the response of the Ag/AlN memristor best agrees with the K^+^ ion channel. We observed that the K^+^ ion's current‐voltage behavior in the HH‐model exhibits a pinched hysteresis loop and matches our experimentally obtained results. The first cycle sees the memristor's lowest change in current, and each subsequent cycle is nearly double to triple the first cycle's current in Figure 3A. By the fourth cycle, the memristor's I‐V curve appears to stop changing and a steady state is reached. Simulation with the HH‐model equations shows a similar trend as seen in Figure 3B. Moreover, we see that the current of the AlN/Ag memristor climbs to ≈45 nA and is similar to the maximum of the K^+^ ion channel. Comparisons to the ideal Na^+^ ion channel under the same stimuli also exhibited similarities to the experimental results. However, the rate of current growth and the maximum current of the Na^+^ ions significantly exceeded the results that were obtained by the Ag/AlN memristor. Again, this behavior agrees with that of our vertically configured memristor. The voltage‐gated potassium ion channels are normally closed, and open when the cell becomes more positive relative to the extracellular fluid to increase permeability to the potassium ions. In the memristor, we can treat the changing device conductivity as effectively increasing the number of “channels” opening from the increasing presence of Ag nanoparticles in the AlN medium. The applied electric potential will cause “channels” to open more quickly and allow for quicker influx of Ag nanoparticles, which explains why higher voltage pulses are able to stimulate the device more dramatically. Additionally, the memristor device will short‐circuit if all of its “channels” are opened, which matches the expectation for when an external stimulus is applied for too long, or if its magnitude is too large.

We next present how electrode geometry can alter memristor behavior. In a ‘diagonally offset’ configuration (Figure 3E), the top and bottom Ag electrodes are not aligned on the vertical axis and are set apart on the horizontal axis, by 100nm. Only the bottom Ag electrode is deposited with an N_2_‐Ar plasma at varying pressures. The top Ag electrode is deposited with an Ar only plasma at a conventionally low pressure. The thickness of the AlN is the same as the vertically configured device. The design assumption is that filamentation or diffusion‐driven conduction would have to take place in both horizontal and vertical directions. Indeed, it can be shown that there are differences in the STDP trends of the diagonal offset devices that distinguish them from the vertically configured devices. In Figure 3F‐i, we observed that with increasing Ag sputtering pressure, the max synaptic weight change of the device is decreased—which is the opposite of the relationship observed in the vertically configured devices. The magnitude of the maximum synaptic weight change for diagonal devices does not exceed 20% at any of the tested formation pressures—this is significantly less than what was achieved by the vertical devices. Most significantly, the diagonal devices at lower formation pressures have multiple peaks and reverse their behavior under repeated stimulation. This observation suggests that it is possible to trigger long‐term depression (LTD) in the diagonal device by increasing the temporal separations while preserving the firing sequence of the pre and postsynaptic neurons. In other words, we can trigger LTD in the device even if the presynaptic neuron fires before the postsynaptic. A similar trend can be observed for negative temporal separations (postsynaptic neuron fires before the presynaptic). The multiple‐peaking behavior regresses with increasing pressure, and the diagonal device begins to resemble a vertical design with weaker weight changes. Overall, the differences in the STDP trends would suggest that the filamentation process in the vertical devices may be less volatile than in the diagonal devices. The vertical Ag/AlN devices are most directly affected by the timing between when a pre and postsynaptic signal is fired, and less sensitive to the individual stimulus on the presynaptic and postsynaptic neurons. In contrast, the diagonal Ag/AlN devices exhibit greater sensitivity to both the timing as well as the individual stimulus of the pre and postsynaptic neurons.

**Figure 4:**
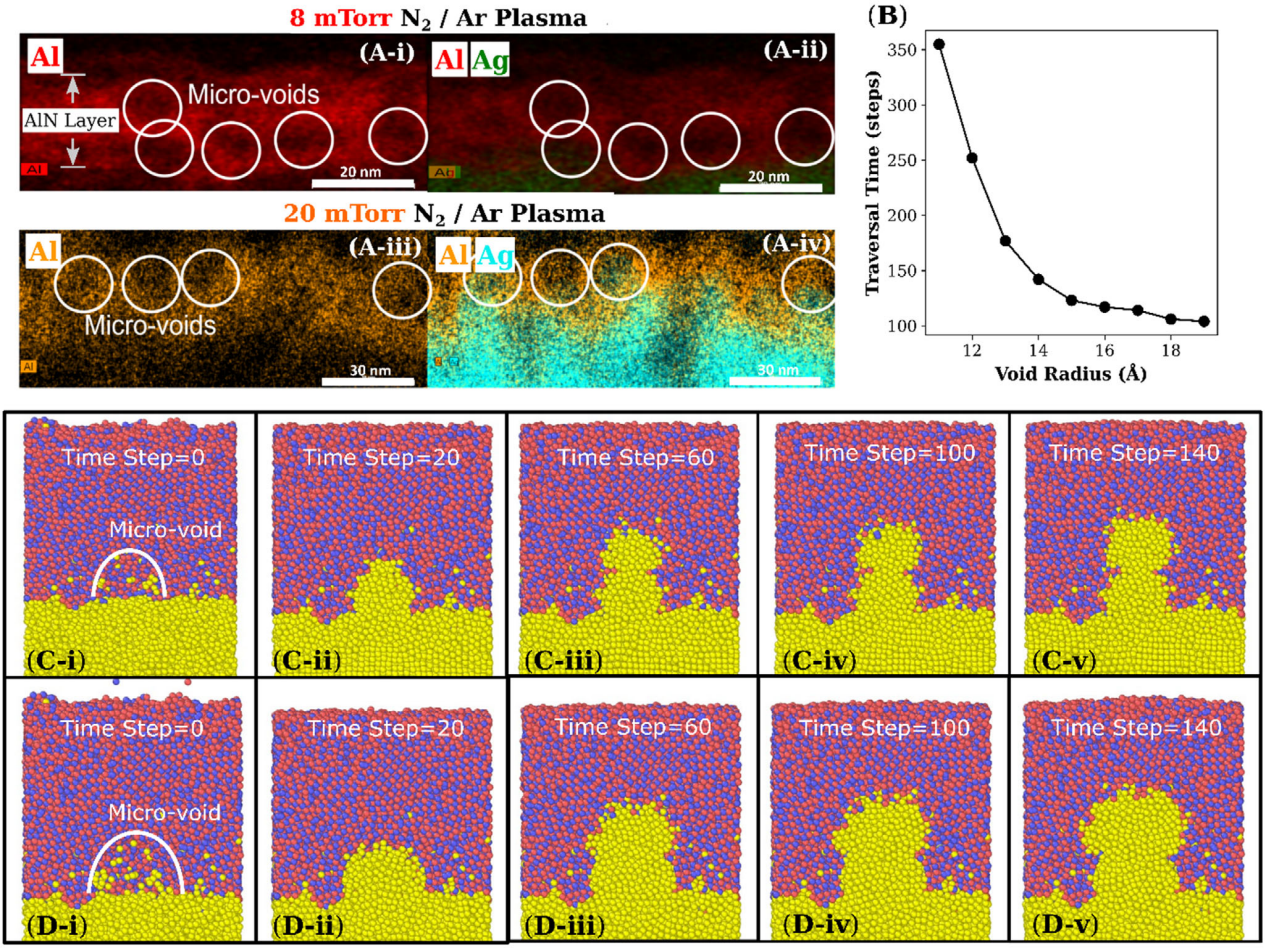
Silver diffusion through micro‐voids A) TEM and EDX microscopy on a Focused Ion Beam milled cross‐section of a 50nm AlN/ 50nm Ag bilayer where the Ag was deposited with 8 mTorr (i) or 20 mTorr N_2_/Ar plasma pressure (ii). The AlN layer contains regions with minimal Al signal, as circled and labeled as micro‐voids, near the interface. In the 20 mTorr sample, in the regions with low Al signal, some Ag signal was detected. The amount of Ag signal intensity was not as high as the Ag layer, indicating that the micro‐voids were only partially filled with Ag. B)The traversal time of silver decreases with increasing micro‐void sizes from 10 to 20 Å radii. Bottom rows: LAAMPs simulated Ag diffusion from the interface into a micro‐void and into 2/3^rd^ of the AlN thickness. The micro‐void in C) was 25 atoms wide and doubled in width in D), where filamentation necking was observed to be absent.

There are several possible drivers of the favorable filamentation and unique plasticity behaviors in the aforementioned results. The first driver of Ag diffusion into AlN can be the presence of micro‐voids within the AlN layer (**Figure** **4**). As seen in the EDX mapping of the cross‐sectional TEM slices of AlN/Ag bilayer of 8 mTorr (Figure 4A‐i, ii) and 20 mTorr (Figure 4A‐iii, iv) each, the AlN layer has missing Al signal with many voids near the interface for the 8 mTorr sample and within the middle of the layer for the 20 mTorr sample. Despite the seemingly larger distance from the interface compared to the 8 mTorr sample, the voids of the 20 mTorr sample are found to contain a Ag signal, although not as strong intensity as the underlying Ag layer, and also with only partial filling of each micro‐void. This indicates that there are Ag nanoparticles within the AlN layer, with the possibility of easy enlargement of particle size. We conducted LAAMPs (Large‐scale Atomic/Molecular Massively Parallel Simulator) based atomic diffusion simulations to understand the effect of micro‐voids on diffusion. We placed a half‐spherical micro‐void in the AlN layer near the interface and applied a positive voltage bias between the top and bottom of the unit cell. We found that as expected, with increasing micro‐void sizes, the diffusion of Ag became more favorable with shorter traversal times from the interface to the surface (Figure 4B). With larger micro‐voids the traversal times become more similar, indicating that there is a limited benefit to increasing the size of large micro‐voids. An examination of the Ag diffusion and filamentation path (Figure 4C) showed that with a small micro‐void of 25 atoms width, the Ag atoms fill the entirety of the micro‐void volume, before the tip of the void breaks to enable Ag to push into the remaining AlN bulk. This results in a filamentation with a narrow neck that acts as the limiting source of the filamentation growth past the micro‐void. With a larger void (Figure 4D) the neck is not present and the width of the filamentation past the micro‐void is larger than the void diameter itself. The reason why the traversal times are similar for a range of large voids is that the filamentation growth becomes more lateral past the void. The results indicate that while the curvature of the Ag nanoparticle filling the micro‐void enables favorable further filamentation, there is a limit to the benefit of introducing larger micro‐voids from greater N outgassing from Ag layer to AlN layer.

**Figure 5:**
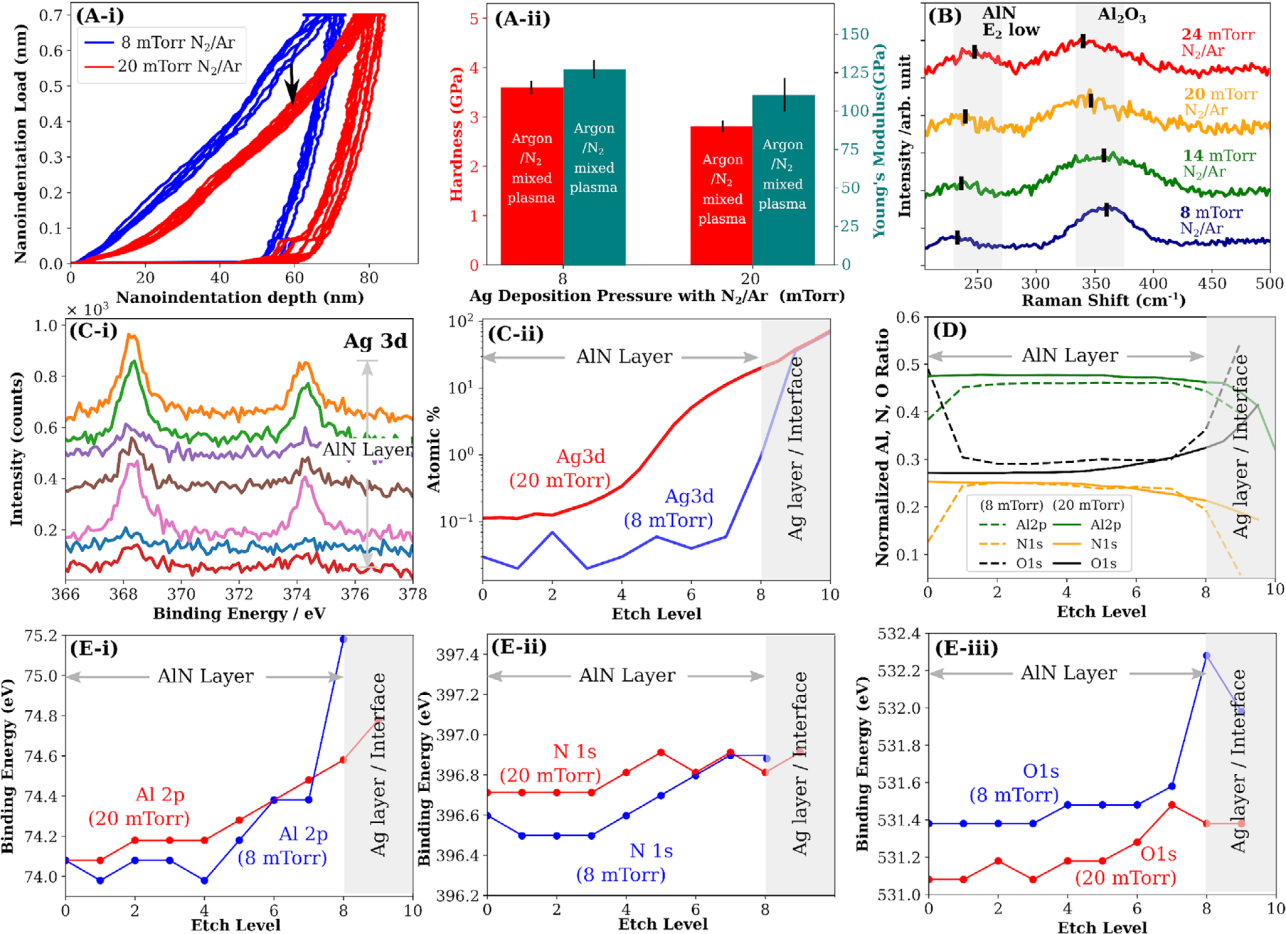
Mechanical strength measurements and X‐ray Photoelectron Spectroscopy depth profiling. A‐i) The nano‐indentation testing of the AlN layer on Ag film deposited with 8 mTorr and 20 mTorr N_2_‐Ar showed the presence of tensile stress from the downward shift in the loading curves. A‐ii) Load‐displacement curves show a 22% decrease in hardness and 14% decrease in Young's Modulus. B) Raman spectra of the top AlN layer with 8 mTorr–24 mTorr N_2_‐Ar deposited Ag underlying layer. A broad peak associated with Al_2_O_3_ is detected, with a peak position decrease indicating lattice strain relaxation; whereas the peak associated with E_2_ low mode of AlN has a peak position increase indicating increasing lattice strain. C‐i) XPS spectra of Ag 3d from the AlN surface (bottom spectra) to the Ag interface (top spectra) for an 8 mTorr N_2_‐Ar Ag deposited sample. Small amounts of Ag were detected in the AlN layer. C‐ii) The Ag atomic concentration within the AlN layer showed higher concentration and deeper penetration from Ag layer for the 20 mTorr associated sample. D) Binding energies of Al 2p (i) and N 1s (ii) from the surface of AlN to Ag interface show similar oxidation state trends between the bulk of the samples, with an increase in the oxidation state of Al at the interface for the 8 mTorr associated sample. D) Al, O, N concentration ratio (normalizing without Ag atomic signal) from the AlN surface to Ag interface. For the 8 mTorr sample, the nitrogen and oxygen concentration at the interface is lower and higher respectively, indicating a higher concentration of interface defects than the 20 mTorr sample. E‐i) The Al 2p binding energy trend indicates the small presence of Al_2_O_3_ at the interface of 8 mTorr sample. E‐ii) The N1s binding energy in the film bulk is higher for the 20 mTorr sample. E‐iii) The oxygen binding energy profiles in the AlN layer show a consistently higher oxidation state for the 8 mTorr sample and a binding energy close to that of passivating aluminum oxide near the interface. The 20 mTorr sample showed a lower oxygen binding energy throughout the AlN layer.

The presence of micro‐voids indicates that at least locally the fracture toughness is overcome by a large stress. The second driver of filamentation can thus be the presence of residual stresses in the AlN layer due to lattice strain. Nano‐indentation hardness measurements^[^ ^34^ ^]^ were carried out on two AlN/Ag films with N_2_ addition and at 8 and 20 mTorr plasma pressures of Ag deposition (**Figure** **5**A). The nano‐indentation Berkovich diamond tip was driven ≈2/3 depth into the AlN layer of 110nm to avoid measuring the composite mechanical properties of the AlN/Ag interface. We found that the AlN layer hardness is substantially decreased by 22% (0.79 GPa) by a higher N_2_‐Ar plasma pressure, and the Young's modulus (within the range of 66–200 GPa expected for amorphous AlN films^[^ ^35^, ^36^ ^]^ was lower by only 13%. The shift downward in the loading curve from 8 to 20 mTorr samples suggests the presence of additional tensile stress in AlN. With the presence of significant tensile stresses, it is expected that the AlN film would be easily exfoliated if not already exfoliated after the deposition process. We carried out a simple tape test to determine the ease of exfoliation of the AlN film, however, the AlN layer of 20 mTorr associated samples remained robust and relatively undamaged. This implies that the high stresses may be locally relieved and interact less strongly with the Ag interface, thus reducing the possibility of exfoliation. To understand the stress distribution, we carried out Raman spectroscopy on the as‐deposited AlN/Ag films at various Ag deposition pressures. In all bilayers with N_2_ included in Ag deposition at all pressures, the presence of AlN with Al_2_O_3_ was detected at the Raman shifts of ≈250 and 360 cm^−1^ respectively. We found two changes in the peak positions of these two phases‐ the Al_2_O_3_ associated peak continually had a negative Raman shift difference by up to a 4 cm^−1^ difference between pressure intervals; and the AlN associated peak had a positive Raman shift difference of ≈2cm^−1^ difference between pressure intervals. This indicates an increasing amount of compressive residual stress in the AlN phase and a counteracting increasing tensile stress relief within the Al_2_O_3_ phase, with increasing N_2_‐Ar Ag sputtering pressures. The E_2_ low mode stress coefficient (Δω  = *K^II^* σ_*film*_) for AlN was shown to be *K^II^* = 0.72 or 0.94 cm^−1^/GPa, which suggests a stress of 6.78–8.85 GPa for the AlN phase. The large differences in Al_2_O_3_ peak position suggest a large relief of compressive stress (estimated to be 14.8–17.4 GPa based on a stress coefficient^[^ ^37^ ^]^ of 1.1–1.3 cm^−1^/GPa) such that there is a large net tensile stress within the AlN. By comparison, it has been shown that a 55nm AlN thin film on silicon by conventional reactive sputter deposition has a residual stress of 249 MPa^[^ ^38^ ^]^ to 300 MPa with observable cracking.^[^ ^24^ ^]^ In all, both hardness measurements and Raman spectroscopy suggest that there is an overall net tensile stress with negating compressive stress, such that the tensile stress overcomes the fracture toughness locally through the creation of micro‐voids, without causing the entire film to exfoliate or crack.

The source of the residual stress can be discerned through XPS‐etched depth profiling, which can determine with better accuracy the quantity of Ag diffusion profile and the oxidation states of the various elements. The 50nm AlN/ 50nm Ag bilayer film was excavated with a Bi^+^ ion beam with a depth interval of 2 ± 2 nm. Trace signals of Ag 3d (≈0.05% at) were detected the moment the ion beam excavated the AlN surface of an 8 mTorr N2/Ar plasma pressure associated AlN/Ag bilayer sample (Figure 5C‐i). The atomic concentration profiles of Ag 3d between 8 mTorr and 20 mTorr samples (Figure 5C‐ii) showed an order of magnitude increase in trace Ag from the surface of the AlN and a high Ag concentration just beyond the interface. The oxidation state of the trace Ag 3d was found to be associated with the metallic state‐ which indicates that adventitious oxidation of the Ag within AlN was hindered even a few weeks after their deposition. This is likely due to the oxidation of AlN, with ≈30% atomic concentration within the AlN layer (Figure 5D). The bulk of the 8 mTorr and 20 mTorr samples are determined to comprise Al_0.45_O_0.30_N_0.25_ and Al_0.43_O_0.27_N_0.25_ with an oxygen‐rich/nitrogen‐poor interfacial region (denoted as 2 etching intervals where the Al signal is minimal). As expected, the increase in N_2_‐Ar pressure during the underlying Ag layer deposition dramatically increases the amount of N in the interfacial region and increases the Al to O ratio in the rest of the bulk. This indicates that a higher N_2_‐Ar pressure of the Ag deposition causes the subsequently deposited AlN to be less favorable to adventitious oxidation, likely due to reactive N outgassing from the Ag layer displacing any oxygen that reaches the interface. The lower oxygen content at the Ag interface for the 20 mTorr sample may also indicate a higher concentration of oxygen vacancies relative to the 8 mTorr sample. A high vacancy concentration has been linked to more facile Ag diffusion through the high atomic defect sites. We note that as mentioned in the prior TOF‐SIMS discussion, the etching beam in XPS can also drive elements ahead of the beam deeper into the bulk, which can account for the over‐representation of Al, O, and N in the interfacial region.

At the interfacial region of the AlN/Ag bilayer of 8 mTorr pressure, the Al 2p oxidation state (Figure E‐i) is similar to that of a passivating Al_2_O_3_ surface formation on an aluminum film with the binding energy of Al rapidly approaches an averaged value associated with Al (IV) (74.8eV) and Al (VI) (74.2eV). For the 20 mTorr pressure associated sample, the Al oxidation state at the interface is higher and lies in the upper limit of the Al_2_O_3_ and AlN binding energy range. The higher binding energy of Al at the interface of 20 mTorr sample is likely due to the better coordination of the Al cations with surrounding O or N anions, which can increase the screening effect of the anions. Because poor coordination at the interface can lead to weaker mechanical coupling between the two layers, this can result in a mismatch in the deformation behavior of the two films (arising from the mismatch in Young's modulus of Ag ≈83 GPa^[^ ^39^ ^]^ and Al_0.57_ O_0.23_N_0.19_ ≈178GPa^[^ ^40^ ^]^). The two layers that are associated with a lower Ag sputtering pressure may be able to deform more independently of each other, reducing the buildup of residual stress in the AlN layer. Conversely, the less abrupt changes in the oxidation state of Al and the higher Al oxidation state in the bulk can indicate stronger interaction with the interface of the 20 mTorr sample. While the oxidation state of N in the bulk of the AlN layer for 8 mTorr and 20 mTorr samples are similar with a small ± 0.1eV difference (Figure 5E‐ii), the oxidation state of the O element in the 20 mTorr sample shows a consistently lower binding energy than that of the 8 mTorr, at the lower limit of the oxidation state of O 1s in AlON. A more negatively charged O^2‐^ ion would indicate lattice distortion to compensate for the negative charge. The contrast in the N and O oxidation binding energy trends thus suggests that in the 20 mTorr sample, the Al‐O and Al‐N coordination is weaker and stronger respectively. We thus attribute the Al_2_O_3_ phase residual stress relief to the decrease in binding energy of O1s and the residual stress increase to the increase in binding energy of Al 2p and N 1s which results in lattice straining.

**Figure 6:**
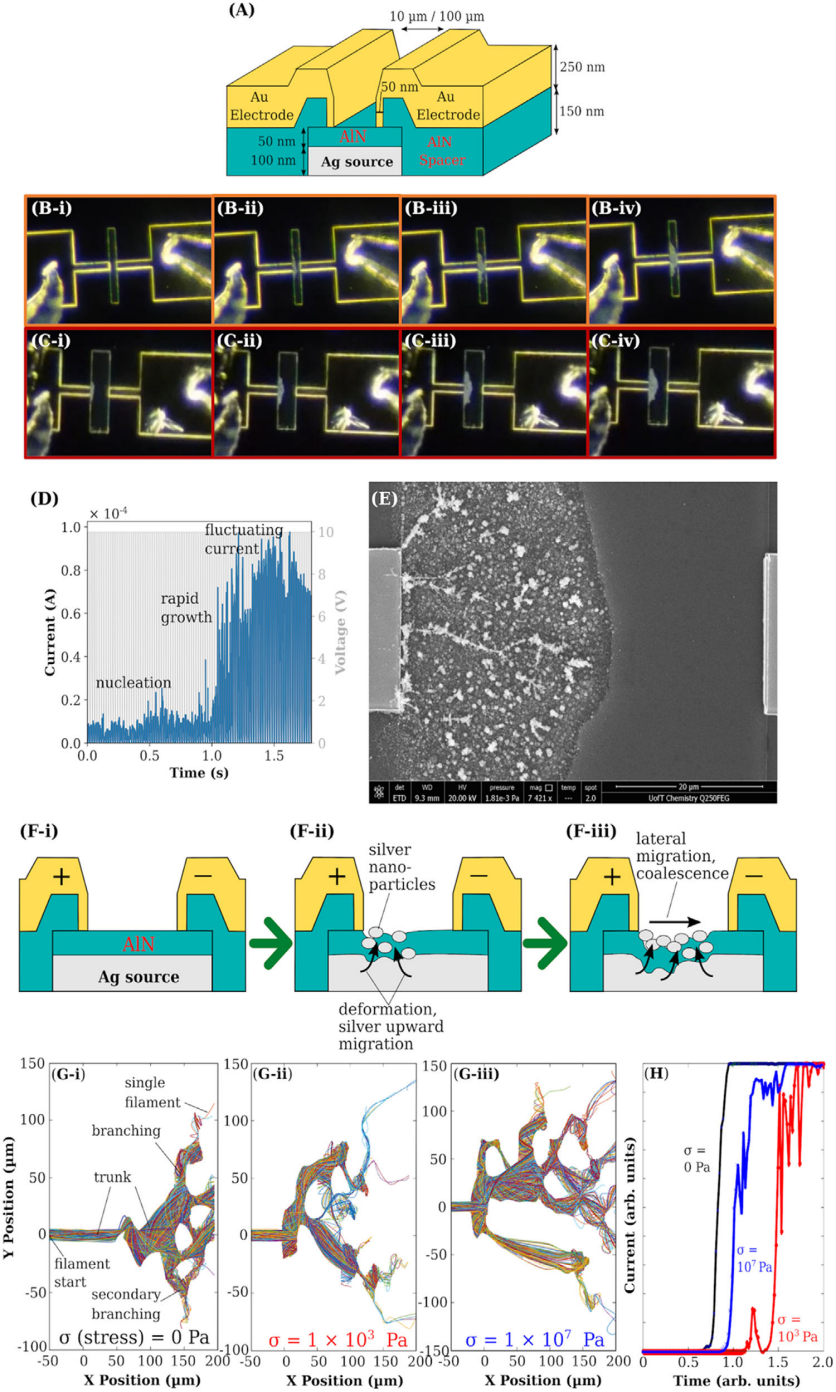
Lateral filamentation growth under high‐speed camera recording, proposed mechanism, and dendritic growth model. A) Laterally configured memristor with 10 µm or 100 µm gap between the gold overlaying electrodes. The underlying Ag layer deposited with N_2_‐Ar at high pressure is defined as the filamentation source. B,C) The lateral silver growth at 10 µm and 100 µm gap, where i) shows emerging Ag once a bias is applied, ii) formation of a Ag region, iii) widening of the Ag region, and iv) no visible change in the Ag region. D) The current growth profile of the 100 µm gap lateral memristor under a 10V pulse train. E) The filamentation dendrite network of the 100 µm gap device. F‐i) Cross‐sectional illustrations of a possible mechanism of lateral filamentation, as determined from EDX profiling of the dendrite network. ii) Ag is drawn from neighboring regions to accumulate and break through the AlN layer at the positive electrode edge. iii) This is followed by coalescence into dendrites. Ag is continually drawn from the region between dendrites to feed the lateral growth. G) Dendritic growth simulations under varying amounts of residual stress from 0 (i) to 10^7^Pa (iii). The dendrite formation consists of a thick trunk emerging from the start‐point; initial branching that connects both ends of the simulation cell; and secondary branching, which can occur as single filaments that increases connectivity and interconnectivity. At higher stresses the secondary branching increases interconnectivity and thicknesses. F) Simulated current behavior from the dendrite growth shown in G). A similar rapid growth before reaching saturation current, and fluctuating current at saturation, is observed in the case of high stress, as shown in H). The current growth behavior and dendrite morphology of 10^9^ Pa closely resemble the experimental results of (D) and (E).

We find that the favorable Ag diffusion and filamentation environment that is presented can enable lateral memristive effects. The lateral filamentation can occur at low switching voltage values that are typically associated with silver diffusion across 2D sheets populated with defects. With a lateral memristor device where the AlN distance between electrodes range is 10–100µm, respectively (**Figure** **6**A), a current growth behavior can be seen to occur over a few seconds (Figure 6B,C). In the first second, Ag was seen to emerge from the positively biased gold electrode, followed by a rapid extension toward the negatively biased gold electrode. At the latter stage, the Ag filamentation widens across its base and Ag densification increases. This approximately matches the current growth behavior, with the low current in the first second associated with (**B‐i**) and (**B‐ii**) and rapid rise to 2/3 of the maximum current from 1.0 to 1.5 s associated with (**B‐iii**), and the subsequent fluctuating currents associated with (**B‐iv**). Interestingly, the current remains low when the silver begins to extend toward the opposing electrode, which suggests that it is a percolation stage where the silver network is not well‐defined and the charge has to hop from Ag particle to particle. The subsequent silver densification and widening in the filamentation region drive the rapid growth in current, whereby the current fluctuation suggests a constant breaking and reforming of the filamentation network. The filamentation in both devices is observed to proceed in three stages‐ the nucleation stage, the rapid growth stage, and the filamentation widening stage.

To understand the filamentation process better, we conducted SEM‐EDX on various spots in the area of lateral filamentation for both 10 µm and 50 µm lateral gap memristors after pulse testing. We positioned the EDX probe beam over the edge of the positive electrode, over the filament, in the areas near the filamentation, as well as the areas where the filamentation did not reach. Because of the low detection sensitivity of nitrogen and oxygen, we compare only the metal atomic ratios of Al and Ag. In the areas that were not disrupted by the filamentation and at the positive electrode, the Ag/(Al+Ag) ratio (denoted as Ag ratio) is 0.6 with 0.4 atomic fractions associated with Al. We thus associate any changes from this ratio standard as deviations from the sub‐surface composition of the as‐prepared device. As expected, the Ag ratio is higher than the standard at 0.68–0.77 directly on the filament. However, at the regions near the filaments, the Ag ratio is lower at 0.29–0.43. For the 50 µm lateral gap device, no well‐defined filament was observed, and the area in front of the positive electrode comprises a distribution of large and small Ag particles.

The Ag ratio directly on the particles is also relatively high at 0.54–0.57, but drops to 0.12–0.22 in the areas between particles. In all, the low Ag content in the gaps between the dendrites and the higher Ag content of the dendrites themselves, as compared to the as‐prepared areas before filamentation, suggests that silver is being drawn from the silver layer toward the filament as it grows. As silver diffuses upward and breaks through the surface of the AlN, silver from the neighboring region diffuses toward the filament and increases its coalescence. As the filament extends toward the negative electrode, more Ag in the larger area diffuses toward the filament, causing the AlN surface to break. For the larger gap memristor, the pattern of Ag distribution suggests that minimal coalescence of the filament took place, but that the larger Ag particles arise from the local diffusion of Ag. Because no filamentation was formed, conduction may occur through percolation of Ag particles, which would require more Ag diffusion from the areas between filaments. This would explain the extremely low concentration of Ag between particles and filaments. Joule heating has been shown^[^ ^41^ ^]^ to be limited in silver dendrite formation as the formation of a thin layer of insulating silver oxide layer at parts of the filament network, can reduce Joule heating and minimize further oxidation. In all, the low concentration of silver in regions between the filament dendrite branches, as well as the higher concentration of Ag on the filaments than the unaffected AlN/Ag film, indicate sub‐surface lateral diffusion of silver beneath the AlN layer.

Given that dendritic formation is easily observed by microscopy, we can model the dendritic formation as a function of time using an Ordinary Differential Solver to determine the net force on the source of Ag particles under an electric field. The movement and attraction between Ag particles are modulated by drag forces, an interfacial potential to form clusters, a pinning potential, and a deformation (exfoliation) potential. We then introduced a simple residual stress term, associated with stresses induced during device deposition, into the model as a constant parameter. The force that the residual stress exerts on the filament is proportional to the length of the filament. Ag nanoparticles that enter the AlN layer will exert greater strain on the structure, and hence a greater force is applied to the filament. The dendritic growth over time at varying stresses is shown in Video S1 (Supporting Information). Additionally, the dendrite and current growth at stress levels (10^2^, 10^6^, 10^9^ Pa) are shown in Figure S10 (Supporting Information). To further explore the role of operational conditions on filament morphology, we extended our simulations to include the effect of compliance current, as discussed in Figure S11 (Supporting Information).

The final states of the dendritic formation at stress levels of 0, 10^3^, 10^7^ Pa are shown in Figure 6G with the associated current growth behavior shown in Figure 6H. At zero stress, the filament emerges as one trunk before widening and subsequently branching to reach the opposite (right) end of the simulation cell (where current saturates and remains constantly saturated). The branching occurs as a single filament that reaches the right end of the cell before additional filaments emerge to widen the branches. Even after the dendrite connects at both ends, secondary branching still emerges on both sides of the dendritic networks, all emerging near the right end of the cell. These secondary branches increase the interconnectivity of the dendritic network and also increase the number of filaments connecting to the opposite end of the cell.

With increasing stress at an intermediate level of σ  = 1 × 10^3^ Pa, the trunk becomes shorter, and the initial branching is slightly narrower than the trunk but becomes increasingly narrower as the network extends to the opposite end of the simulation cell. The initial branch reaches the end of the cell as a thick filament. With a shorter trunk, the secondary branches emerge at various distances along the simulation cell. The secondary branches near the trunk end emerge as a thick filament. The secondary branches that emerge near the right end of the cell emerge as single filaments and travel in the opposite direction of the filament growth before looping around to extend toward the right end of the cell. The reversed travel and looping generate fluctuations in the saturated current. The widening of the branches partially occurs by single filaments emerging from one branch and immediately looping back to the branch. The single filament secondary branches initially form as a line of scattered particles before more particles fill the gaps to form a continuous line.

At a high‐stress level of σ = 1 × 10^6^ Pa, multiple branches emerge initially and continually branch as single filaments near the left end (initial start) of the cell. Some single filaments loop back to the initial branch network and grow thicker as interconnecting branches. This generates a dense dendrite network near the start of the cell. Some of the single filaments that travel to the opposite right end of the cell may loop a number of times as they develop. Other single filaments that emerge later contribute to the thickening of the forward single filaments. An important observation is that, similar to the secondary branching in the intermediate stress case, the initial branching emerges as scattered particles forming the filament, before more particles connect the particles to form a continuous line. This causes the current to rise without reaching the saturated current level. Similar to the intermediate stress case, the subsequent current fluctuations occur as additional filaments loop toward the end of the cell or back to the branch. At very high‐stress levels of 10^7^–10^9^ Pa, both branching and densification occur simultaneously, which leads to an increase in the current behavior such that the threshold time is shorter than that of 10^3^ Pa case. The current growth profile and dendrite morphology associated with 10^7^ Pa bears a close resemblance to that of the experimental lateral growth results in Figure 6D,E, which validates our assumption that residual stress is an important factor. In all, the introduction of residual stress is likely to increase the chance of charge hopping, branching near the initial start, and dendrites forming backward to the initial start or increasing dendrite interconnectivity.

### 2.1 Significance of Synaptic Behavior

We hypothesize that increasing residual stress increases the density of paths that branch from the main filamentation, which enables the silver nanoparticles to traverse the AlN medium along multiple energetically equivalent pathways as shown in Figure 6G. This leads to the observed differences in the STDP response and these physical changes could lead to more robust artificial neuron units. With the use of longer periods between the pre and postsynaptic signals, the relaxation of the filament may become comparable to how quickly filamentation can occur. This effect can slow down the filamentation in one direction which would be reflected as a decrease in the synaptic weight change, as can be observed in the interval of 15–50 µs in Figure 3F‐i. We noticed that the magnitude of the negative peak that is reached is not equal to the magnitude of the initial peak because the existing filament has not completely degraded. This observation may be a result of how the presence of micro‐voids in AlN changes the path of least resistance. In the vertical device, increasing the formation pressure does not change the shortest path to the electrode—the micro‐voids make it easier for Ag^+^ to traverse in the vertical direction. In contrast, micro‐voids in the diagonal device seem not to be distributed diagonally. Instead, at higher formation pressures it may become easier to traverse vertically and laterally than diagonally. A non‐diagonal conductance pathway is suggested by the fact that the STDP learning trend in Figure 3F‐ii,iii closely resembles a vertical device with a weaker synaptic change response. In the lower pressure device, a re‐spiking that is observed after 50 µs in Figure 3F‐i suggests a sensitivity to the individual stimulus. Since the timing is sufficiently long to lose the “cause and effect” correlation of pre and postsynaptic stimuli, the application of individual stimuli must induce filamentation. The spike signal applied at the presynaptic and postsynaptic nodes is each contributing to driving the Ag nanoparticle to form a filament. Increasing the temporal spacing between the application of each stimulus leads to the time scale becoming comparable with the filamentation relaxation and reduces the magnitude of the synaptic weight change, as observed in vertically configured devices. However, these results suggest that the filamentation relaxation in diagonal devices is such that the diffused Ag nanoparticles are in closer proximity to the filament and electrode than in vertical devices. Hence, the application of the postsynaptic stimuli can actually drive the diffused Ag nanoparticles to recombine with the filament structure and appear as weak secondary peaks in the synaptic weight change plot (**F‐i** and **F‐ii**). Eventually, the separation between the stimuli is sufficiently large that the filamentation relaxation effects dominate and the synaptic weight change approaches zero (**F‐iii**). An intriguing application of this behavior is in the action potential formation process in biological neurons. The sign change in synaptic weights of the diagonal devices is akin to hyperpolarization, which prevents a real neuron from undergoing action potentials in rapid succession. Hence, it would be feasible to reverse memristor conductivity through only varying pre and postsynaptic timing (i.e. without changing the firing order) and enable continuous stimulation and inhibition in a networked array.

In neuromorphic computing applications, a model of STDP behavior can assist in the training of a spiked neural network (SNN). SNNs are one possible architecture for neuromorphic computing systems and are intended to mimic the human brain as closely as possible. Training of the SNN can be achieved by updating the synaptic weights via mechanisms like STDP. In reported work in the literature^[^ ^42^, ^43^, ^44^ ^]^ demonstrations of SNN performance in unsupervised learning or supervised learning approaches have shown performance comparable to that of artificial neural networks (ANNs). Simulations performed by^[^ ^45^ ^]^ to train a SNN utilized two different types of STDP learning rules to demonstrate how the outcome of SNN performance is tied to the STDP function that is used. Different neural coding schemes^[^ ^35^ ^]^ which encode the input will also influence the performance of the SNN. The results shown in previous works^[^ ^43^, ^45^, ^46^ ^]^ have demonstrated the capacity of SNNs trained only with a STDP learning rule and present the feasibility for transferring these networks onto physical hardware. Currently, an implementation of a SNN utilizing only CMOS circuitry incurs a high production cost to mimic the STDP update rule of a single memristor.^[^ ^47^ ^]^ The AlN/Ag memristor's known STDP learning rule in the vertically configured device can be most closely classified as an additive STDP rule). The significance of a known STDP update function intrinsic to the device points to the potential of fabricating plastic synapses with single memristors and integrating them into CMOS networks for a hardware deployable SNN.^[^ ^47^ ^]^ Even prior to deployment, simulations of SNN performance with the memristor's known STDP response can be performed to assess its usability. Moreover, different STDP learning rules can influence the training and performance of the SNN as presented in the survey of STDP.^[^ ^44^ ^]^ Our work has shown that simply altering the position of the Ag source of the AlN/Ag memristor can produce more complex STDP learning rules^[^ ^48^ ^]^ that make it possible to implement a greater variety of potential SNN training regimes.

## 3 Conclusion

Metal filamentation and its neuromorphic behaviors under an unconventional sputtering approach show various unique drivers for low‐powered memristors. We found that non‐reactive sputter deposition of the underlying silver layer with a combined nitrogen‐argon plasma dramatically lowers the voltage bias threshold for memristance, whereas modifying the reactive sputter deposition of the overlaying aluminum nitride does not enable memristance. In addition, we found that modifying the sputtering pressure of N_2_‐Ar delivers dramatic changes in memristance performance, whereas minimal observable changes were seen with modifying the N_2_/Ar flow ratios and power. We showed that one possible driver for facile silver filamentation is the introduction of residual stress from lattice straining of the adventitious aluminum oxide component of the nominally sputtered aluminum nitride. From our nano‐indentation measurements, the net stress is observed to be as high as 30 MPa, which would normally cause exfoliation. We found that with increasing Ag sputtering pressures, the oxidation state of oxygen in the bulk of the AlN layer decreases, whereas the oxidation state of nitrogen increases, which indicates that the adventitious oxidation of AlN occurs to a lesser degree with higher pressures. The stress relief in the AlN bulk expected from increasing the nitrogen content in AlN is countered by an increase in lattice straining in the Al_2_O_3_ phase within the AlN layer. This form of localized stress may explain why the AlN layer shows no signs of exfoliation or cracking, whereas high residual stress throughout a film is typically relieved by peeling off from the interface. In our devices, the residual stresses appear to also induce the formation of micro‐void defects, which were evidenced by regions with minimal Al signal in cross‐sectional EDX analysis. These micro‐voids were found to be partially filled with silver, thereby providing a local source of silver diffusion.

In the higher range of sputtering pressures used, the STDP and memristive changes are less dramatic, likely owing to the negligible benefit of larger micro‐void sizes (due to the tendency of the filaments to widen), and smaller increases in net residual stress. Transitioning from lower to higher sputtering pressures results in a significant change in the STDP and memristive behavior in the form of a larger net conductivity (synaptic weight) change and faster current growth, respectively. These results suggest that there is a threshold pressure at which changes induced by sputtering pressures in the device structure become significant enough to alter its memristive characteristics. Furthermore, a laterally offset electrode orientation alters the most energetically viable path length for filamentation and alters the STDP behavior to exhibit multiple maxima and minima. With increasing pressure, however, the STDP response loses its multiple peaks and begins to resemble that of a vertically configured device‐ this implies that residual stress potentials reduce the lateral component of filament growth and enhance its vertical component. In both cases, the memristor current growth behavior is analogous to what is expected of potassium ion current in a biological action potential. Given these results, the AlN/Ag memristor offers a potential platform for applications as an artificial neuron in neuromorphic computing devices. Further, this technique is likely to be generalizable to a wide variety of metals and metal alloys‐ opening up filamentation‐based memristors to incorporate materials beyond silver.

In all, inducing larger‐than‐atomic sized defects and residual stress associated with lattice strain are new drivers, which are unique apart from the conventional atomic vacancy‐driven model, to generate a wide variety of memristive behaviors at low voltage biases.

## 4 Experimental Section

### Sputter Deposition of Ag and AlN and Au films

The sputter‐deposition system was a KJL cryo‐pumped multi‐target magnetron RF sputtering facility with a base vacuum of ≈ 2 × 10^−6^ Torr. The deposition of the first Ag layer of 100–200. nm thickness was carried out at a power of 150 watts with N_2_:Ar flow ratio of 12:8 sccm and at pressures described within. Subsequently, deposition of the 50 nm AlN layer was carried out by reactive sputtering of an Al target with N_2_‐Ar plasma at power of 400 W with N_2_:Ar flow ratio of 9:11 sccm and pressures of 3–4 mTorr.

### Pulse IV Measurements

The pulse‐train tests were composed of a series of identical square pulses. 20 consecutive 2 V pulses were applied with a pulse duration of 6 ms, and a timing of 0.2–700 ms between pulses. The relaxation tests were comprised of an initial long switching pulse followed by a train of short and low‐voltage read pulses. The relaxation time was defined as the time required to reach a low resistance (on) state, and the delay time was the time required for the device to return to a high resistance (off) state. The duration of the switching pulse used during relaxation testing ranged from 2.5 to 50 ms, and the voltage ranged from 1 to 10 V. The duration of the read pulses ranged from 0.25 to 25 ms, and the voltage ranged from 0.0075 to 1 V, with a timing of 10 ms between pulses. The number of read pulses ranged from 10 to 40. From these tests, the relaxation time appears to vary between 0.1 and 10. ms, and the delay time varies from 0.02 to 1.5 ms.

### LAAMPS Simulation

Our molecular dynamics simulations, conducted with the Large‐scale Atomic/Molecular Massively Parallel Simulator (LAMMPS), were designed to investigate the diffusion mechanics of silver (Ag) atoms through micro‐voids within an aluminum nitride (AlN) layer. The simulation utilized a 3D box measuring ≈7.5 × 7.5 × 15 nm^3^, providing a sufficiently large space to model the deposition processes and subsequent atom migration. Both NVE and NVT ensembles were used to regulate the dynamics of the atoms.

The initial phase of the simulation was focused on the deposition of a silver layer. In order to model the sputter deposition process as accurately as possible, the substrate layers were constructed by simulating the process. This was accomplished using LAMMPS' “fix deposit” command which allowed for the periodic release of one or more atoms with a specified energy distribution every few picoseconds (much like during a sputter deposition process). A thin layer of fixed silver atoms was initially used, followed by the introduction of additional atoms one by one to construct a layer that fills, the height/width of the box and stands ≈5 nm tall. To accurately model the interactions between silver atoms and achieve a realistic formation of the^[^ ^49^ ^]^ deposited layer, an EAM potential commonly used to model Ag atoms was employed. This facilitated the formation of an Ag layer with intended characteristics and dimensions.

Following the silver deposition, the deposition of Al and N atoms proceeded to form the AlN layer, with the “fix deposit” command employed once again. A tersoff potential^[^ ^50^ ^]^ was used to accurately represent the interactions among Al‐Al, N‐N, and Al‐N atoms, allowing for the realistic formation of the AlN layer.^[^ ^24^ ^]^ An EAM potential was used to model the interaction between the Al and Ag atoms,^[^ ^51^ ^]^ and a basic Lennard–Jones potential was used to model the Ag‐N interactions. The deposition continued until the layer reached ≈7.5 × 7.5 × 7.5 nm^3^ in size. To simulate the experimental observation of micro‐voids and their role in Ag diffusion, a half‐sphere of atoms within the AlN layer, centered at the Ag‐AlN interface, was strategically removed. This modification allowed the bottom few atoms of the AlN layer to be fixed in space–preventing the entire layer from floating upward due to the silver diffusion process.

Since the Ag atoms were modeled as charged ions, an external electric field (‘efield’ command in LAMMPS) was applied to investigate the diffusion dynamics of silver atoms through the AlN layer, particularly how the micro‐void parameters affect the diffusion process. The results of this command are shown in Figure 4 in the main text.

### Dendritic Growth Simulation (Details in Supplementary Information)

The Ag diffusion and, consequently, the memristor switching mechanism were modeled using a numerical simulation performed with MATLAB. Inspired by the model from Wang^[^ ^52^ ^]^ et al, our model uses a built‐in ODE solver to calculate the net force on each Ag nanoparticle to determine the morphology of the resulting dendrites. The differential equation of particle movement to be solved is:

(1) $$F_{net}=F_{p}+F_{a}+F_{d}$$

(2) $$\eta \frac{dx}{dt}=-\frac{\partial U}{\partial x}-\eta C_{d}v_{x}^{2}+\alpha \frac{V\left(t\right)}{L}$$

(3) $$\eta \frac{dy}{dt}=-\frac{\partial U}{\partial y}-\eta C_{d}v_{y}^{2}$$

where *F_p_* was a summation of the interfacial, defect potential, and deformation potential forces, *F_a_* was the force of the applied potential, and *F_d_* was the drag force. Additionally, η is the viscosity, α was the induced charge and *C_d_* was the drag coefficient. The potential energy *U* is determined by both the interfacial forces between silver particles and the potential energy landscape in the AlN film.

The positions of the silver particles were specified by *x_i_* and *y_i_* where *i* was the nanoparticle label. The motion of the particles in *x* and *y* directions (where the start and end positions of the particles were on the left and right side of the simulation cell‐ particles travel generally along the *x* direction and widen/branch out in the *y* direction) can be shown as:

(4) $$\eta \frac{dx}{dt}=-\frac{\partial U}{\partial x}+\alpha \frac{V\left(t\right)}{L}-\eta C_{d}v_{x}^{2};\eta \frac{dy}{dt}=-\frac{\partial U}{\partial y}-\eta C_{d}v_{y}^{2};$$

The first term on the right side of the equation directs the particle toward the point of minimal potential energy at a velocity determined by the local gradient of the potential energy. The drift term, $\alpha \frac{V(t)}{L}$ was the result of the electric field *E*  =  *V*(*t*)/*L* applied to the device. The drag term $-\eta C_{d}v_{y}^{2}\;$ and $-\eta C_{d}v_{x}^{2}$ represents the drag force on each particle as it moves through the AlN medium.

### Resistance Modeling of Dendrite Growth

The overall resistance of the device was governed by the tunneling resistance between pairs of nanoparticles. In Ohm's law, *V*  =  *IR*(*w*) where *R*(*w*) is the resistance of the device while in a particular state w. This state variable evolved in time when an external electric bias was applied. The dynamical properties of *w* was governed by the second equation: $\frac{dw}{dt}=f\left(w;i\right)$.

The state‐dependent resistance of the device was simulated via Monte Carlo methods. At each timestep of the nanoparticle diffusion process, the migration of n_e_ electrons (randomly placed at the location of Ag nanoparticles) was simulated. This was executed by using a sub‐timescale (of *t_s_* steps) to track the tunneling of each electron. At every timestep, each electron could either tunnel to another location or stay put. The probability that electron i will tunnel to location j was given by:

(5) $$p\left(e_{i}\to p_{j}\right)=\frac{{\left(R_{t}.e^{d_{ij}/\lambda }\right)}^{-1}}{\sum_{i=1}^{n_{e}}\sum_{j=1}^{n}{\left(R_{t}.e^{d_{ij}/\lambda }\right)}^{-1}}$$

where λ was the effective tunneling length of the electron, *d* was the distance between electron *i* and location *j*, and *L* was the length of the device. Each electron's next location was sampled from this distribution. The electron was therefore most likely to tunnel to a close‐by particle or not tunnel at all. After *t_s_* sub‐timesteps, the percent of electrons that made it to the end of the device is calculated. A scalable approximation of the current through the device was given.

### Residual Stress Addition to Dendrite Growth

The residual stress was added as a constant parameter to the model. This was to account for combined effects that may have altered the device's structure during the sputtering process, exfoliation of gases into the AlN layer, and diffusion of Ag nanoparticles into the AlN structure. The residual stress was seen in the dendrite growth as the movement of nanoparticles into the AlN layer strained the overall structure inducing a force onto the filament. In a simplified 2D model, it was assumed that particles could move vertically or laterally, with stresses acting in a vertical and horizontal direction—no shear was considered. Hence, the strains along the horizontal and vertical direction could be found by

(6) $$\varepsilon_{xx}=\frac{\sigma_{xx}}{E}-v\frac{\sigma_{yy}}{E}$$

(7) $$\varepsilon_{yy}=\frac{\sigma_{yy}}{E}-v\frac{\sigma_{xx}}{E}$$

where was the strain in the horizontal direction, ε_*yy*_was the strain in the vertical direction, σ_*xx*_ was the horizontal stress, σ_*yy*_ was the vertical stress, *E* is Young's Modulus for AlN, and ν is Poisson's ratio. In the model, Young's Modulus was set to 323.5 GPa and Poisson's ratio to 0.23 which were representative values of nominal AlN. The stress on a single particle was calculated as

(8) $$\sigma_{xx}=\frac{F_{app}+F_{e}}{2\Delta x^{2}}+\sigma_{res}$$

(9) $$\sigma_{yy}=\frac{F_{e}}{2\Delta y^{2}}+\sigma_{res}$$

where σ_*xx*_ was the horizontal stress, σ_*yy*_ was the vertical stress, *F_app_* was the force from the applied potential, *F_e_* was the electrostatic force between two particles, σ_*res*_ was the residual stress, Δ*x* was the width of the filament in x and Δ*y* was the width of the filament in y. It was noted that the simplifying assumption was made that the surface that was seen by the application of the stress was a square with either dimension of Δ*x* or Δ*y*.

The computed strains were then used to update the particles’ current positions for the next time step in the simulation. A cubic function was used to account for particles near the edge of the filamentation traveling further compared to the ones that make up the bulk of the filament—this resulted in more movement breaking away from the filament's edge rather than in its main body.

## Conflict of Interest

The authors declare no conflict of interest.

## Supporting information



Supporting Information

Supplemental Video 1

Supplemental Video 2

## Data Availability

The data that support the findings of this study are available from the corresponding author upon reasonable request.
