# Supplementary material for: Residual Stresses and Micro‐voids Propel Metal Diffusion for Filament‐Based Memristors
Source: Adv Sci (Weinh). 2025 May 9;12(22):2416305. doi: 10.1002/advs.202416305 (PMC12165104; doi:10.1002/advs.202416305)
Supplement: Supplementary file 1 — Supporting Information [file ADVS-12-2416305-s003.docx]

**Supplementary Information to “Residual Stresses and Micro-voids Propel Metal Diffusion for Filament-based Memristors”**

**Time of Flight-Ion Spectroscopy (TOF-SIMs) of Oxygen and Nitrogen in Bilayers of AlN over Ag without N2 at 8mTorr and with varying N2/Ar pressures during deposition of underlying Ag layer.**
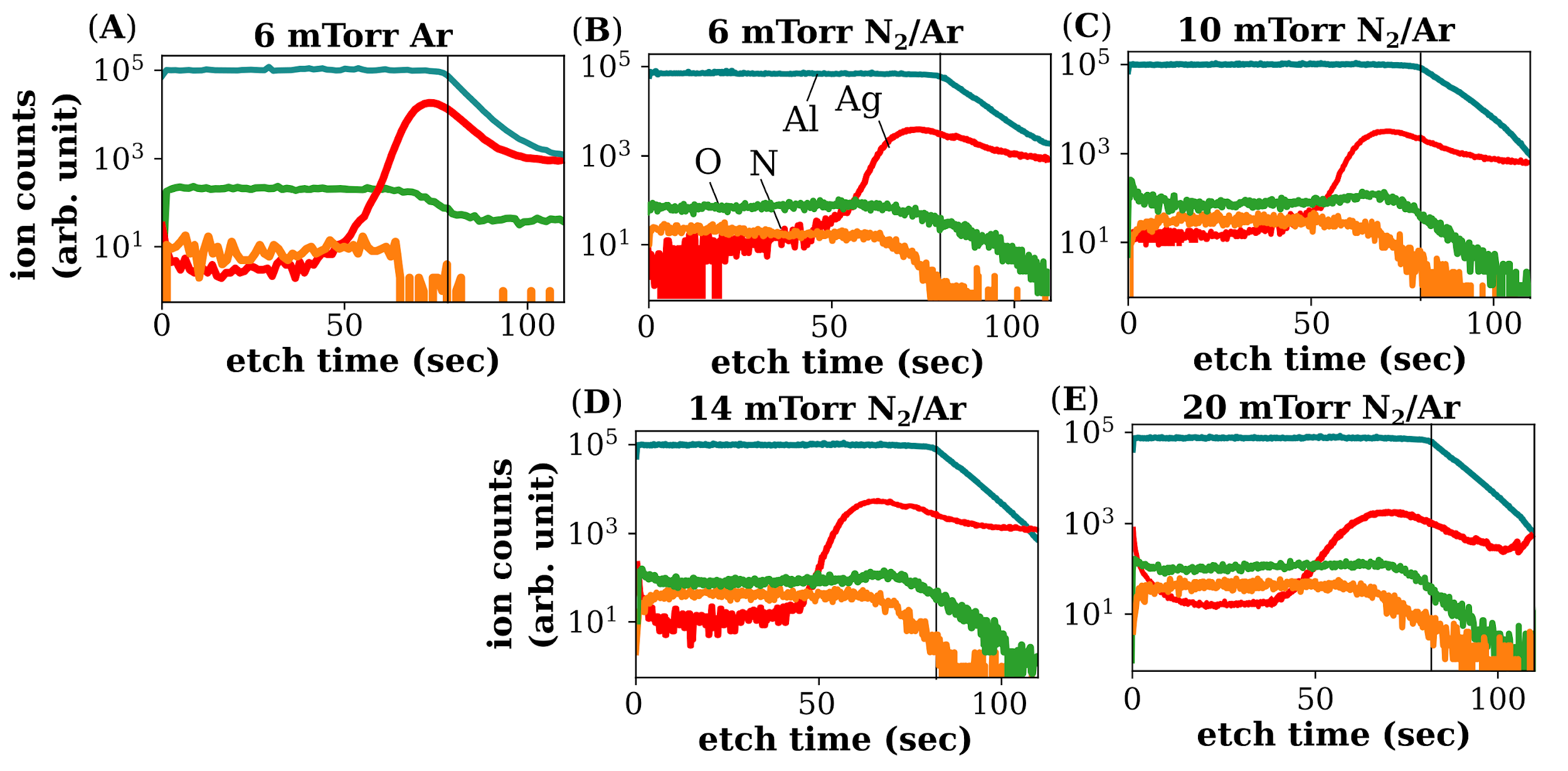


**Figure S1**. (**A**) TOFSIMs of 6mTorr Argon without nitrogen plasma in Ag deposition. Very low concentrations of silver in most of the AlN layer. The O and N profiles are shown in (**A-E**) where adventitious oxygen in the Ag layer is reduced when N2 is introduced. N element is minimized at the interface (A) but extends into the Ag layer with the introduction of N2 and higher pressures.

Because the etching ion beam causes a collision cascade of atoms within a few nanometers ahead of the beam, this results in atomic mixing as some of the Al atoms are buried or re-deposited below the interfacial level[19]. The Al signal decays instead of abruptly ceasing at the interface, thus causing the measurement artifact. Another commonly seen TOFSIMs artifact arises at the interface where the amount of Ag signal peaks, which can be associated with positive charge accumulation at the dielectric AlN layer that causes preferential sputtering of the silver when the ion beam excavates the interface - thus leading to over-representation of Ag [20], [21]

**
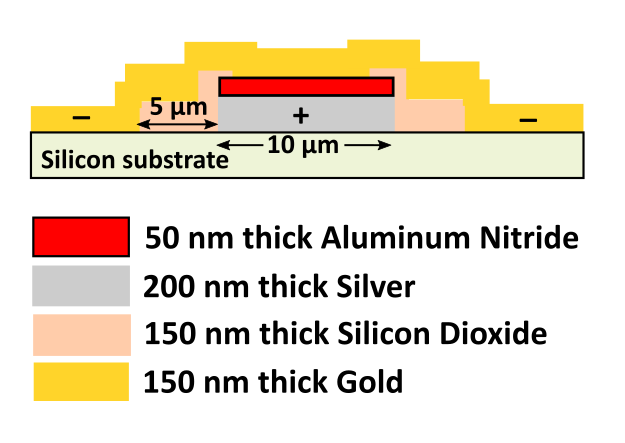
**

**Figure S1**. (**F**) Cross sectional image of main **Figure 1A**, from the perspective of the positive gold electrode. The width and length of the SiO_2_ spacer is 5μm and 15 μm respectively. The width and length of the memristor active layers (AlN/Ag) is 10 μm and 5 μm respectively.

**SEM/BSE (back-scattered electron) cross-sectional images of AlN on 14mTorr N2/Ar deposited silver layer.**
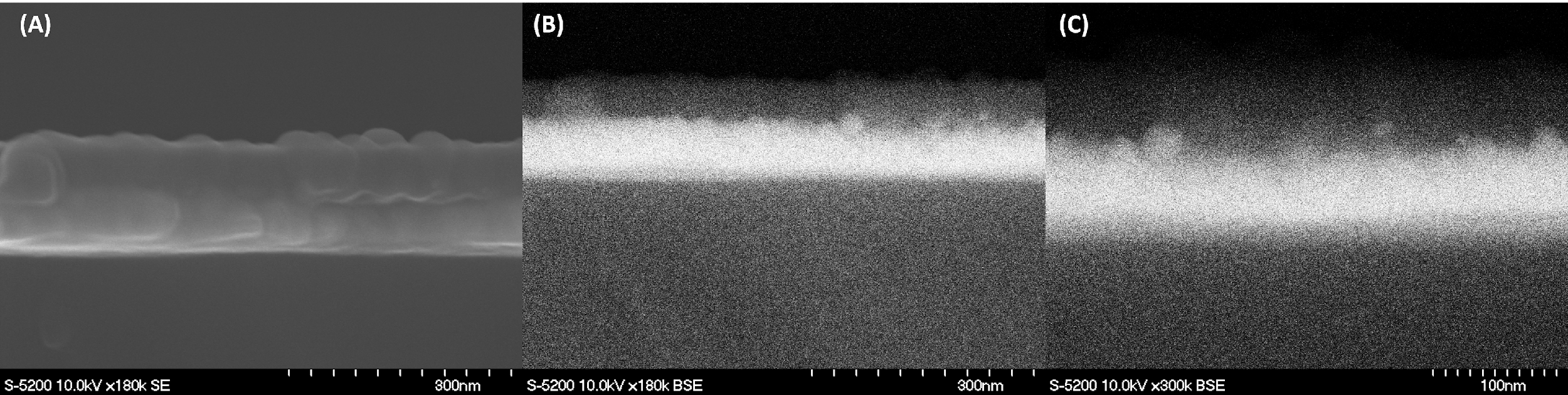


**Figure S2**. (**A**) Cross sectional SEM of a reactive sputtered AlN thin film on Ag layer that has been deposited with 8mTorr N2/Ar plasma. The bilayer film was ion-milled to expose the cross-section. (**B**) BSE mode of the same bilayer shows the presence of silver as a diffused region (left side of image), and small silver particles (right side of image) within the AlN layer and near the interface. (**C**) A zoomed in image of (B) shows a large discrete silver particle at the interface and one ~10nm particle in the middle of the AlN layer


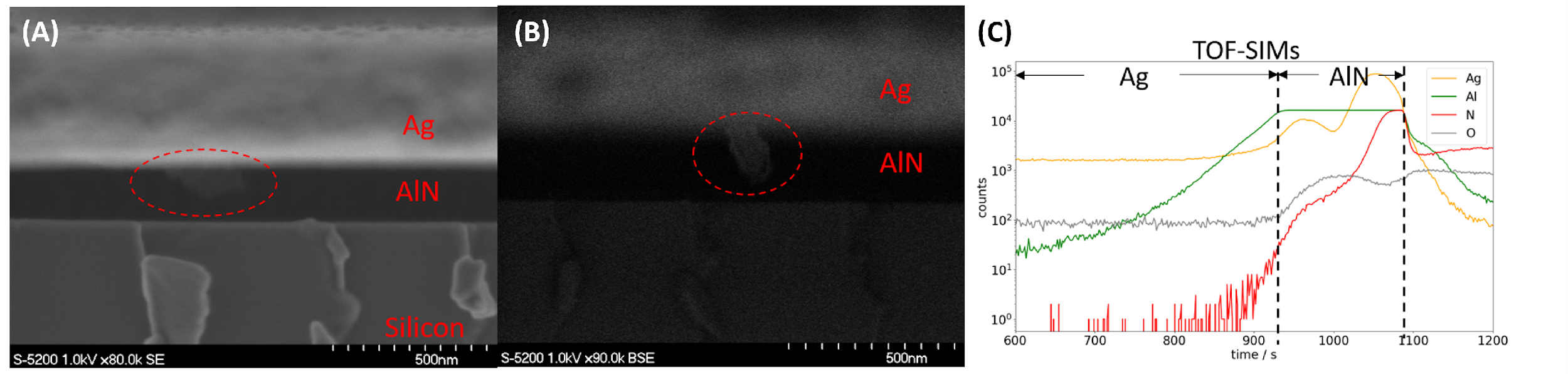
**SEM/BSE Cross-sectional images of Ag (deposited with 8mTorr N2/Ar) on AlN layer.**


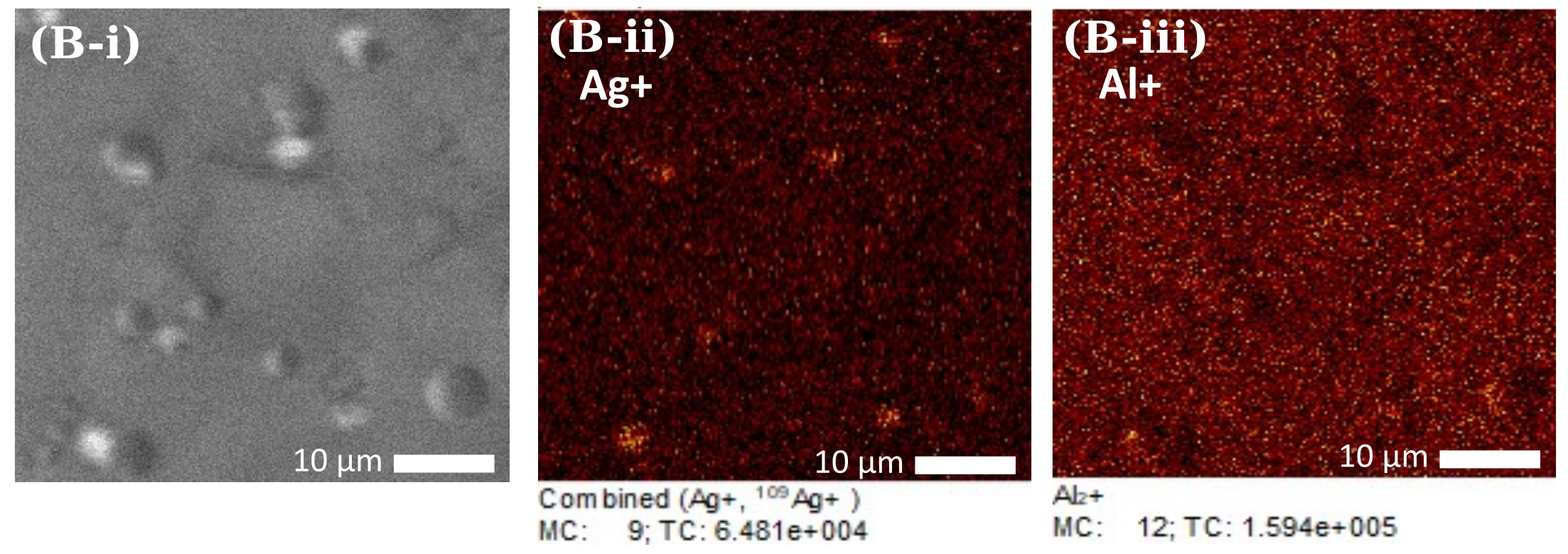


**(D-i)**

**(D-ii)**

**(D-iii)**

**Figure S2D**. SEM image of blisters (i). Ag+ ions seen within the blisters (ii), as detected by TOFSIMS etching of the AlN surface. (iii) shows that the blisters has a surface of Al implying that the Ag is not exposed to the environment.

**
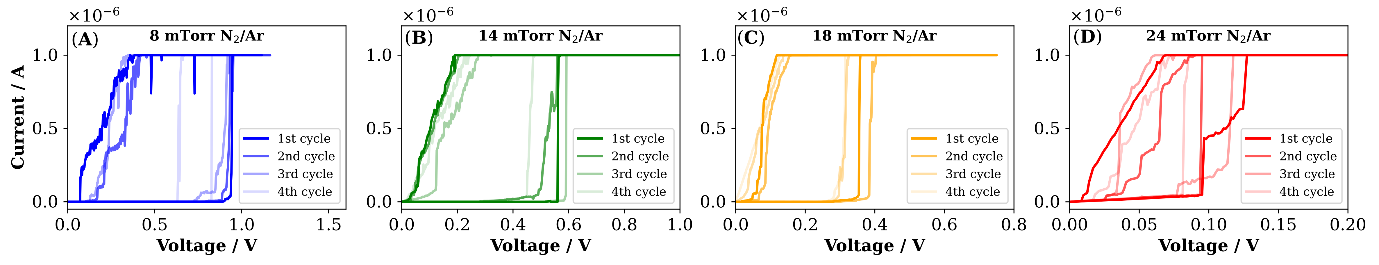
Hysteresis IV Behavior of Memristors with varying N2/Ar pressures during deposition of underlying Ag layer**

**Figure S4**. IV behaviour showed approximately 0.9 V threshold switching voltage with a mixture of N_2_/Ar plasma in Ag deposition. Switching voltages decrease to less than 0.1 V with increasing pressure while maintaining well-behaved hysteresis loops.

**Figure S3**. To test if the N2/Ar sputtering of Ag causes residual N in Ag to only interact upwards, we deposited AlN on silicon with the standard recipe, followed by the deposition of Ag with N2/Ar on top of the AlN. (**A, B**) SEM and BSE of Ag/AlN bilayer shows the presence of silver in the AlN trailing from the interface. BSE of Ag layer shows micro-voids, with one directly above a silver filament. This suggests that the fracture toughness of the silver is exceeded by residual stress in the Ag layer. We note that the BSE mode only shows the contrast between elements based on their atomic mass. It is therefore difficult to see if micro-voids exist in the AlN layer by BSE- hence we turned to cross-sectional TEM. (**C**) TOF-SIMs of the Ag/AlN bilayer. Adventitious oxygen is seen throughout the silver and AlN layer. The etching artifact of TOF-SIMs makes the presence of silver within the AlN layer inconclusive as silver may have been driven into the AlN by the etching beam. However, the negligible presence of N in the Ag layer followed by an increase in concentration at the interfacial region suggests that residual nitrogen remains in the silver and interacts with the underlying AlN layer.

**HH relaxation model**

**Supplementary Figure 5.** The Hodgkin-Huxley equation modelling potassium ion conductance over time fitted to the memristor relaxation after application of 1 voltage switching pulse and a pulse read train. The resting state conductance of the memristor matches that of the memristor following the end of the switching pulse and the steeper drop off in the memristor conductance may be a consequence of the filament stability.


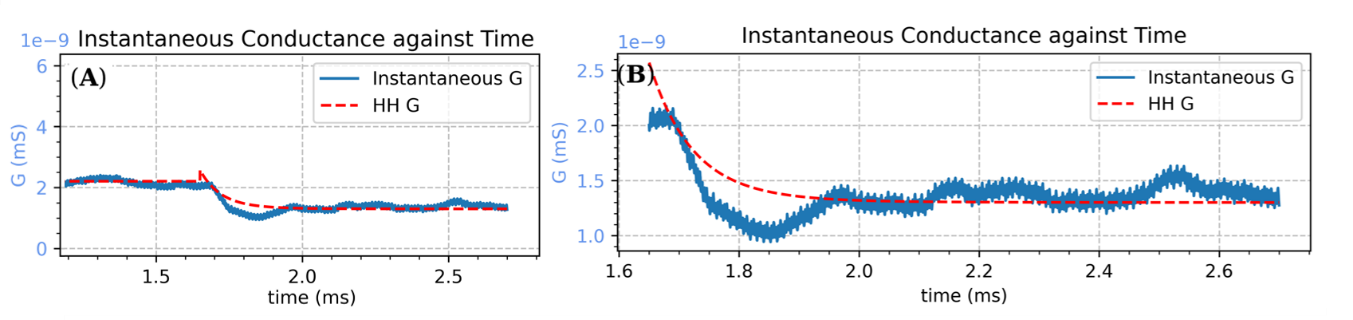
When stimulated by a square voltage pulse, the conductance of the potassium ion channel as simulated according to the Hodgkin-Huxley (HH) equation can also be used to model the conductance of the AlN/Ag memristor. Once the voltage pulse has been shut off, the conductivity of the potassium ion channel will decay. In the memristor, we observe that the relaxation behaviour as probed by the read pulse shows that the device conductance decays and eventually reaches a plateau level. As Supplementary Figure 5 illustrates, the potassium ion conductance from the HH model can be used to describe the relaxation of the device. In the fit, the voltage dependent gating variable *n* at rest potential, n(V_0_), is set to be 0.6, the gating variable at the voltage step potential is n(V_1_) is set to 0.1, the voltage dependent time constant of the gating variable is set to 0.25 ms, and the conductance 0.005 nS. Fits to the Na^+^ ion channel, a combination of Na^+^ and K^+^ ion channels and using the complete HH model were also performed. Only the Na^+^ ion channel behaviour shares some commonalities with the memristor’s result but the Na^+^ relaxation when stimulus was removed was much faster than the memristor’s.

**Figure S5.** The Hodgkin-Huxley equation modelling potassium ion conductance over time fitted to the memristor relaxation after application of 1 voltage switching pulse and a pulse read train. The resting state conductance of the memristor matches that of the memristor following the end of the switching pulse and the steeper drop off in the memristor conductance may be a consequence of the filament stability.

In biological neurons, the conductance of K^+^ at rest and during repolarization is small because the cell is either maintaining its equilibrium or restoring itself to an equilibrium state; hence the reason why the HH-model will approach a conductance value of 0 mS/cm^2^. However, the memristor instead plateaus to a non-zero conductance value. This suggests that the timescale for complete deterioration of the filament may be larger than milliseconds and maintains some concentration of Ag nanoparticles in the AlN layer that keeps the device in a semi-conductive state when unstimulated. In the context of the device’s “learning”, this suggests that there is the possibility for a networked system to determine which connections to strength. The higher the unstimulated memristor conductance, the better it will be at conducting current. Artificial neurons built with these devices can demonstrate potentiation based on their unstimulated conductance — device pathways that are used more frequently would be expected to have a resting state conductance that will be higher.

**STDP measurement**

The function for the presynaptic and postsynaptic waveform is identical and illustrated in [FIGURE]. The waveform is applied to both electrodes of the memristor and differ only in when they are applied. Each postsynaptic spike is separated from the presynaptic waveform by some time interval $\Delta T$—where $\Delta T=T_{post}-T_{pre}$ is the time separation between the first peak of the post and presynaptic waveforms. A spike train of presynaptic and postsynaptic waveform pairs in a time separation sequence of $\Delta T=\{0, 5, -5, 10, -10, \ldots, 60, -60, 70, -70, \ldots, 100, -100\}$ μs is used to stimulate the memristor device (Campbell et al., 2016). A square read pulse with amplitude 0.1 V is sent prior to the start of the pulse sequence and after each $\Delta T$. The response of the memristor to the different $\Delta T$ stimuli is expressed by the synaptic weight change:

$$\Delta w\%=\frac{\frac{1}{R_{2}} - \frac{1}{R_{1}}}{\frac{1}{R_{min}}}\cdot100\%$$

Where $R_{1}$ is the resistance of the device before the pre-postsynaptic pair, $R_{2}$ is the final resistance after the pre-postsynaptic pair and $R_{min}$ is the smallest resistance measured over all $\Delta T$ values used in the spike sequence—$R_{min}$ is for normalization of the weight change percent (Campbell et al., 2016).

**Indentation testing details for all samples**

A Micromaterials Vantage Nanotest System was employed for this testing work. The System is fully compliant to all relevant international nanoindentation standards including ISO14577 and ASTM E2546–07. The system was calibrated with the lowest noise floor and thermal drift rate confirmed in the MechAction Lab. The system and the indenter tip were validated on Fused Silica and Tungsten reference samples according to ISO 14577 standard.
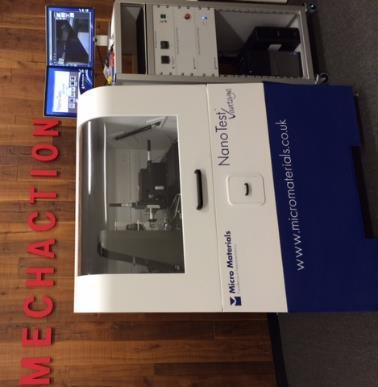


0.7 mN maximum load was selected for so that the maximum indent depth was roughly 60~80nm. Load controlled tests are selected so that the residual stress influence will have a nature response to indentation as the applied load is constant. All other testing parameters were set according to ASTM E2546 and ISO 14577 standards.

**XPS results of 20mTorr sample and comparison of Binding Energy with 10mTorr sample, and XPS results of Samples Without N2 in Ar during Ag deposition.**

**
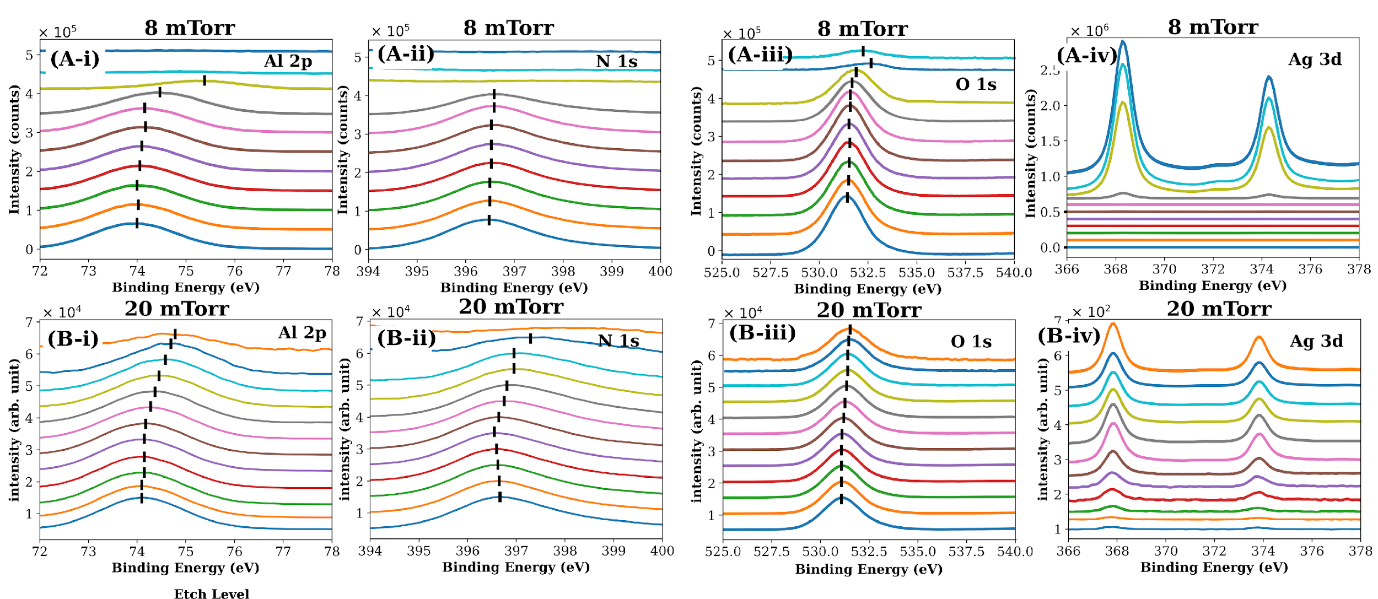
**

The binding energy of Ag shows a lower binding energy for the higher pressure associated sample. Ag is unusual in that a higher oxidation state is associated with a lower binding energy. Ag 3d in Ag metal has a BE of 368.2 eV, whereas Ag 3d in AgO has a lower BE of 367.6 eV
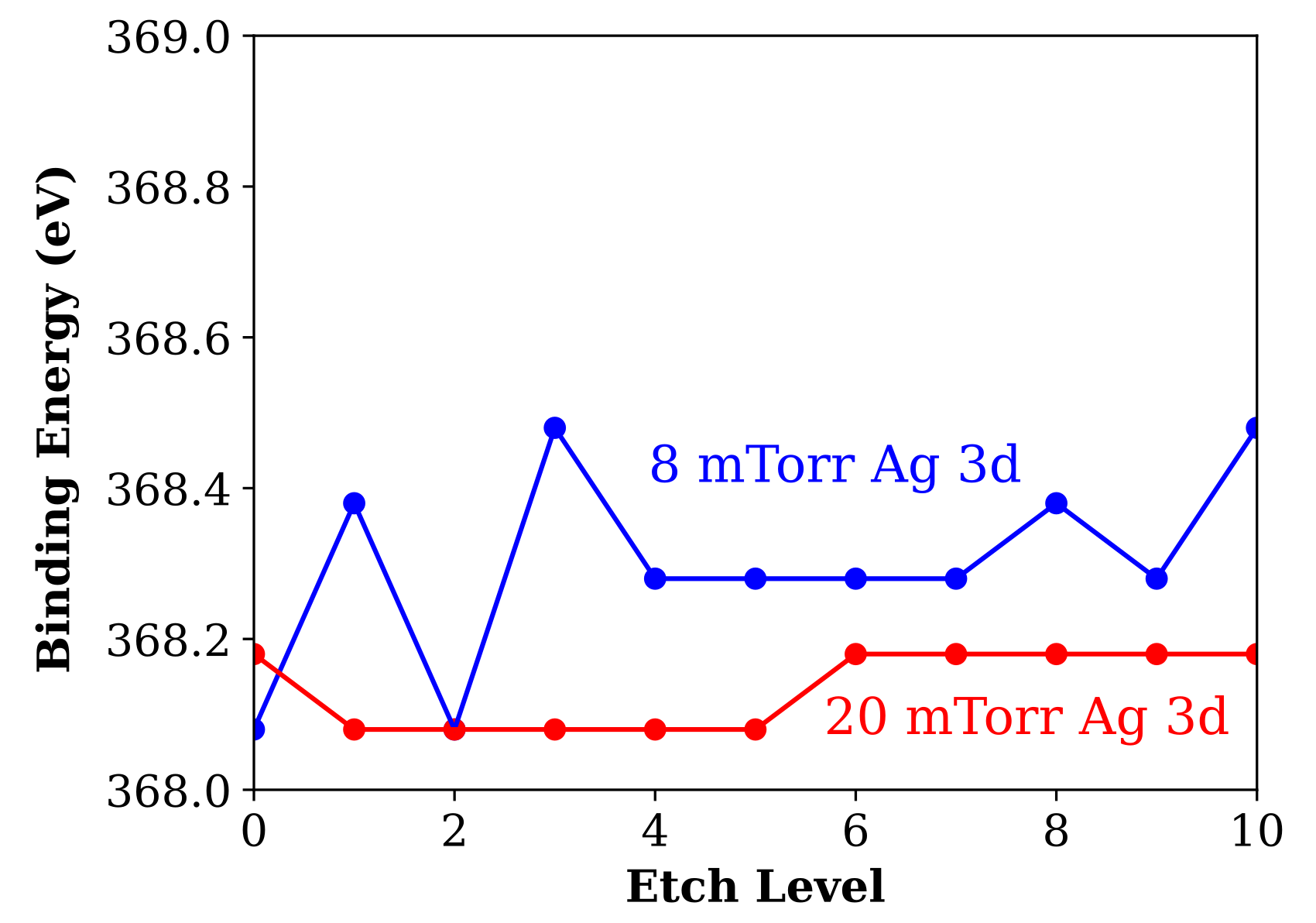


**Figure S6**. The Al binding energy corresponds to Al_2_O_3_ at the immediate interface but rapidly transits to binding energy corresponding to AlN [ref] in the bulk of AlN layer. N and Ag binding energy are lower for the 20mTorr sample. The higher ionization of Ag in the 20mTorr sample associated with a lower binding energy, as an unusual feature of Silver XPS, indicates that the silver migration has an lower ionization threshold due to residual stress (from lattice straining), lower oxygen binding energy in the bulk layer, and higher oxygen vacancies at the Ag interface.

**EDX study of lateral memristors after IV pulse driven filamentation**


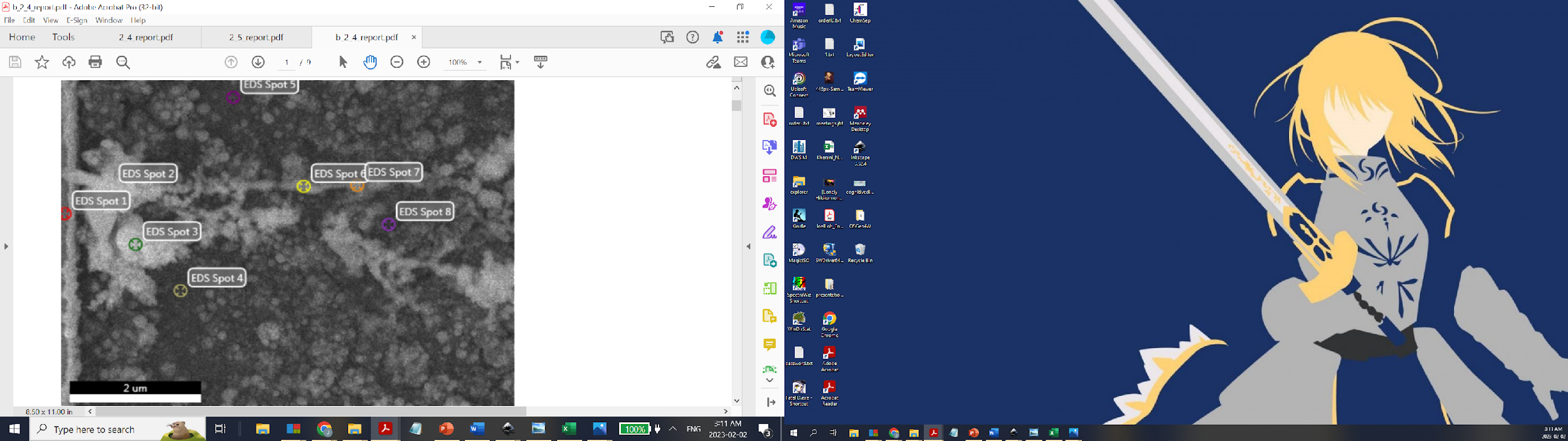


**Figure S7**. SEM image of the lateral filament memristor of 10 μm gap. Various spots on the edge of the electrode (Spot 1), directly on silver filament or particle (spot 2,3, 6, 7) and away/near from filament (spot 4, 5, 8) were taken.


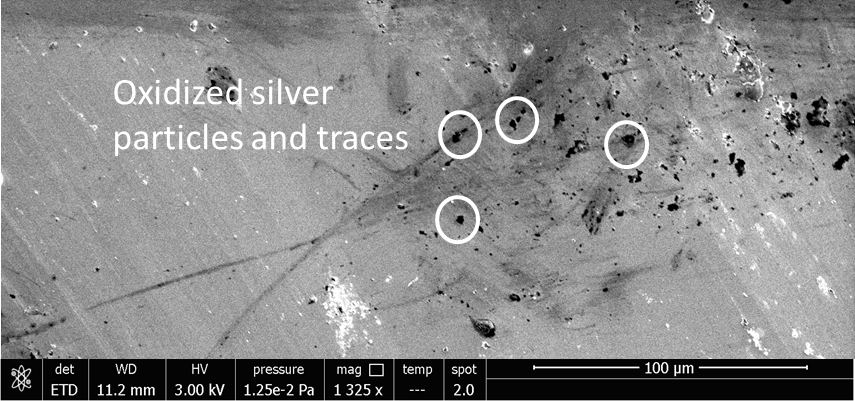


**Figure S7B**. SEM images of AlN/Ag samples that do not show memristive behavior, but show the presence of oxidized silver as dark spots.

| 10 μm gap lateral memristor | | |  |  |  |  |  |  |
| --- | --- | --- | --- | --- | --- | --- | --- | --- |
|  | Electrode | On Filament | | Away from Filament | | On Filament | | Near Filament |
| EDX spot | 1 | 2 | 3 | 4 | 5 | 6 | 7 | 8 |
| Al % a.t | 41.4 | 22.1 | 32.4 | 70.6 | 57.0 | 32.3 | 33.0 | 61.8 |
| Ag % a.t | 58.6 | 77.9 | 67.6 | 29.4 | 43.0 | 67.7 | 67.0 | 38.2 |

| 100 μm gap lateral | | |  |  |  |  |  |  |  |
| --- | --- | --- | --- | --- | --- | --- | --- | --- | --- |
|  | Electrode | In between particles | | | Directly on particle | | Untouched by Filamentation | | |
| EDX spot | 7 | 8 | 6 | 3 | 4 | 5 | 2 | 1 | 9 |
| Al % a.t | 56.6 | 70.3 | 77.8 | 88.0 | 42.3 | 45.5 | 32.2 | 35.5 | 39.9 |
| Ag % a.t | 43.4 | 29.7 | 22.2 | 12.0 | 57.7 | 54.5 | 67.8 | 64.5 | 60.1 |

| EDX Area | Area 1 | Area2 | Area3 |
| --- | --- | --- | --- |
| Al % a.t | 48.4 | 31.9 | 27.3 |
| Ag % a.t | 51.6 | 68.1 | 72.7 |


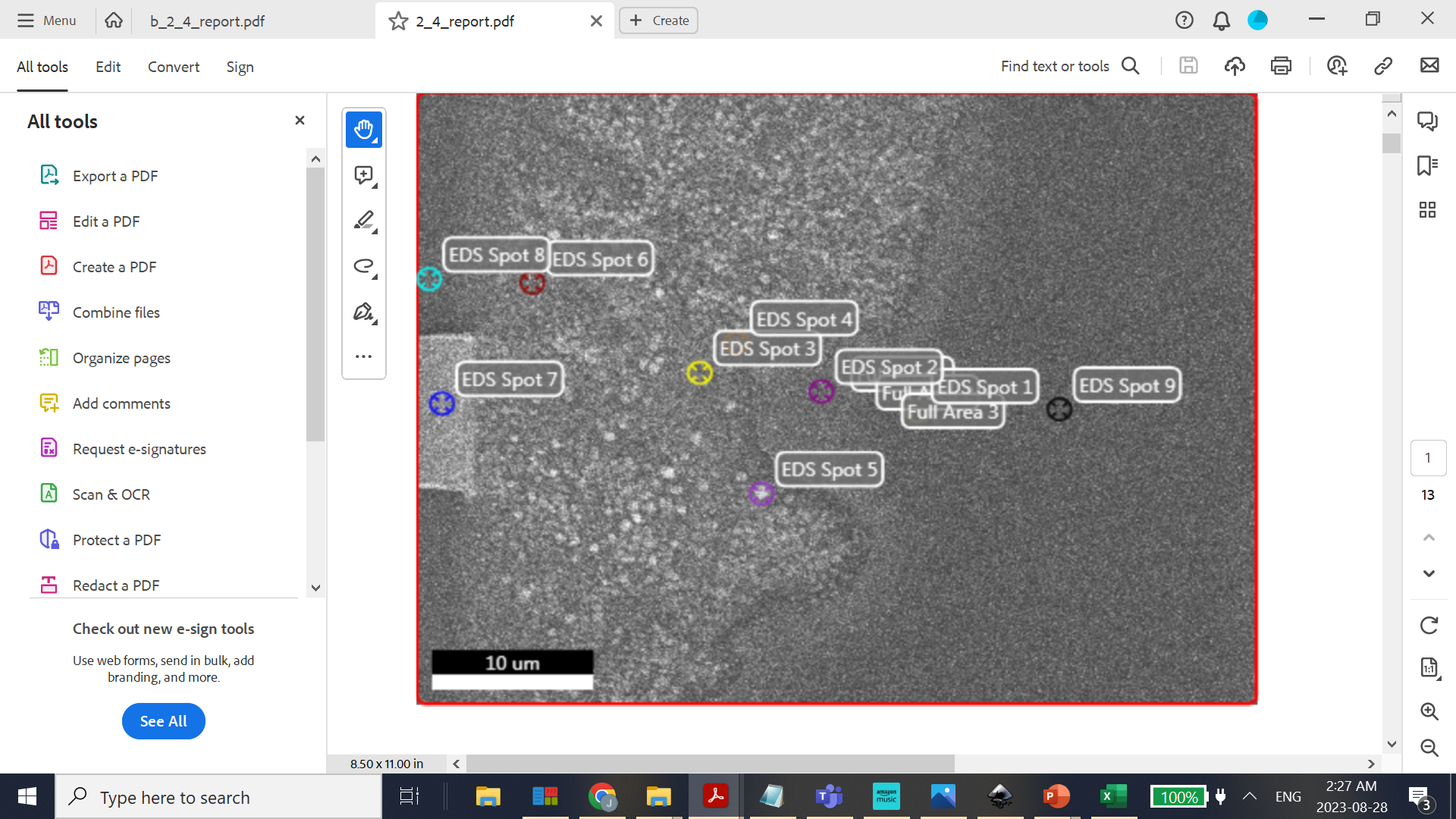


**Figure S8**. SEM image of the lateral filament memristor of 100 μm gap. EDX spots are taken on the electrode (Spot 7), directly on silver particles (spot 4,5), in between particles (spot 3, 6) away from filament (spot 1, 9).

**EDX-TEM cross section of silver filament in tested memristor device**

**
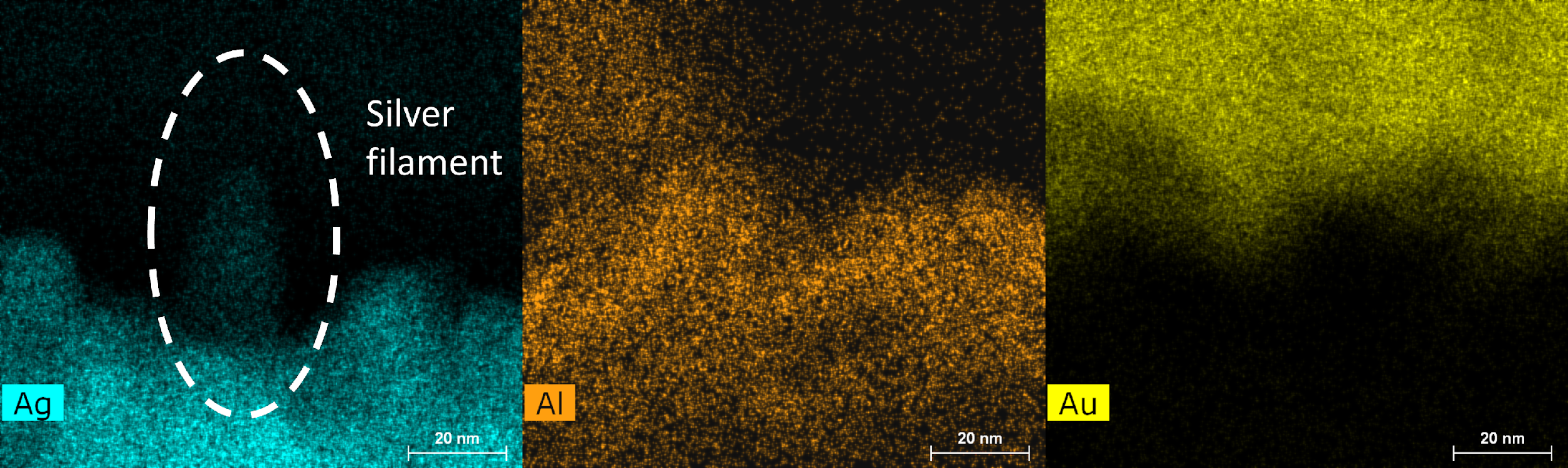
**

**Figure S9**. TEM cross section of an AlN/Ag vertically configured memristor device sputtered with 20mTorr N_2_/Ar after a voltage bias of 0.1 V. The gold electrode is situated on top of the AlN layer. The field of view of SEM image was shifted before capturing the Al and Au signal. Hence the Al and Au image are not aligned with the Ag image. The thick filament of Ag detected showed that silver grows as a ‘finger’ correlating with LAAMPs simulations.

**Dendrite Growth Simulation**

The primary force driving the diffusion results from the potential difference between the electrodes:

The model includes three different types of potentials to determine the force on the particle: $F_{a}=\alpha\frac{V(t)}{L}$

Where $E=V(t)/L$ is the electric field that is applied to the device (L is the length of the device gap between electrodes), and α represents the induced charge on each particle due to the presence of the field. The drag force acting against the particle’s movement as it travels through the AlN medium: $F_{d}=-\eta C_{d}v_{i}^{2}$ where C_d_ is the drag coefficient and *v* is the velocity of the particle *i*. This force has a large effect on the diffusion time and clustering of the nanoparticles. Note that in all potentials: $r_{i}^{2}=\left( x-x_{i} \right)^{2}+\left( y-y_{i} \right)^{2}$

1. The interfacial potential,

$$U_{I}=w_{I}\sum_{i=1}^{n} \sum_{j=1}^{n} e^{\left( r_{ij}-\sigma\right)^{2}/R_{I}^{2}}-e^{\left( r_{ij} \right)^{2}/R_{I}^{2}}$$

Where *r* is the distance between particles *i* and *j*. *w_I_* is the interfacial energy barrier, $\sigma$ is the average radius of the nanoparticles. This potential encourages the nanoparticles to form clusters in order to minimize their interfacial surface energy. In our simulation, this potential also regulates the width and evolution of the dendrites.

The pinning (defect) potential,

$$U_{p}=w_{p}\sum_{i=1}^{n} \sum_{j=1}^{n} e^{\left( r_{ij} \right)^{2}/R_{p}^{2}}$$

Where *w_a_* represents the pinning potential amplitude, m is the number of pinning sites and *R_p_* represents the size of the defect.

The dendrite morphology of the Ag diffusion is primarily a result of the pinning potentials. Physically these pinning sites represent impurities in the substrate or ionic lattice.

1. The AlN deformation potential is a grid-based potential with n_grid_ squares of width 2*R_a_*. Each square starts at state *s_k_*= 0 for 1<*k*<*n_grid_*. As soon as a nanoparticle enters square k, s_K_ = 1. The potential is characterized by:

$$U_{a}=w_{a}\sum_{i=1}^{n} \sum_{k=1}^{n_{grid}} {s_{k}.e}^{\left( r_{ik} \right)^{2}/R_{a}^{2}}$$

Where *w_a_* represents the peeling potential amplitude and *r_ik_* is the distance between particle *i* and centre of square *k*. This potential directs the particles towards the peeled regions of AlN.

**Dendrite Growth Simulations**


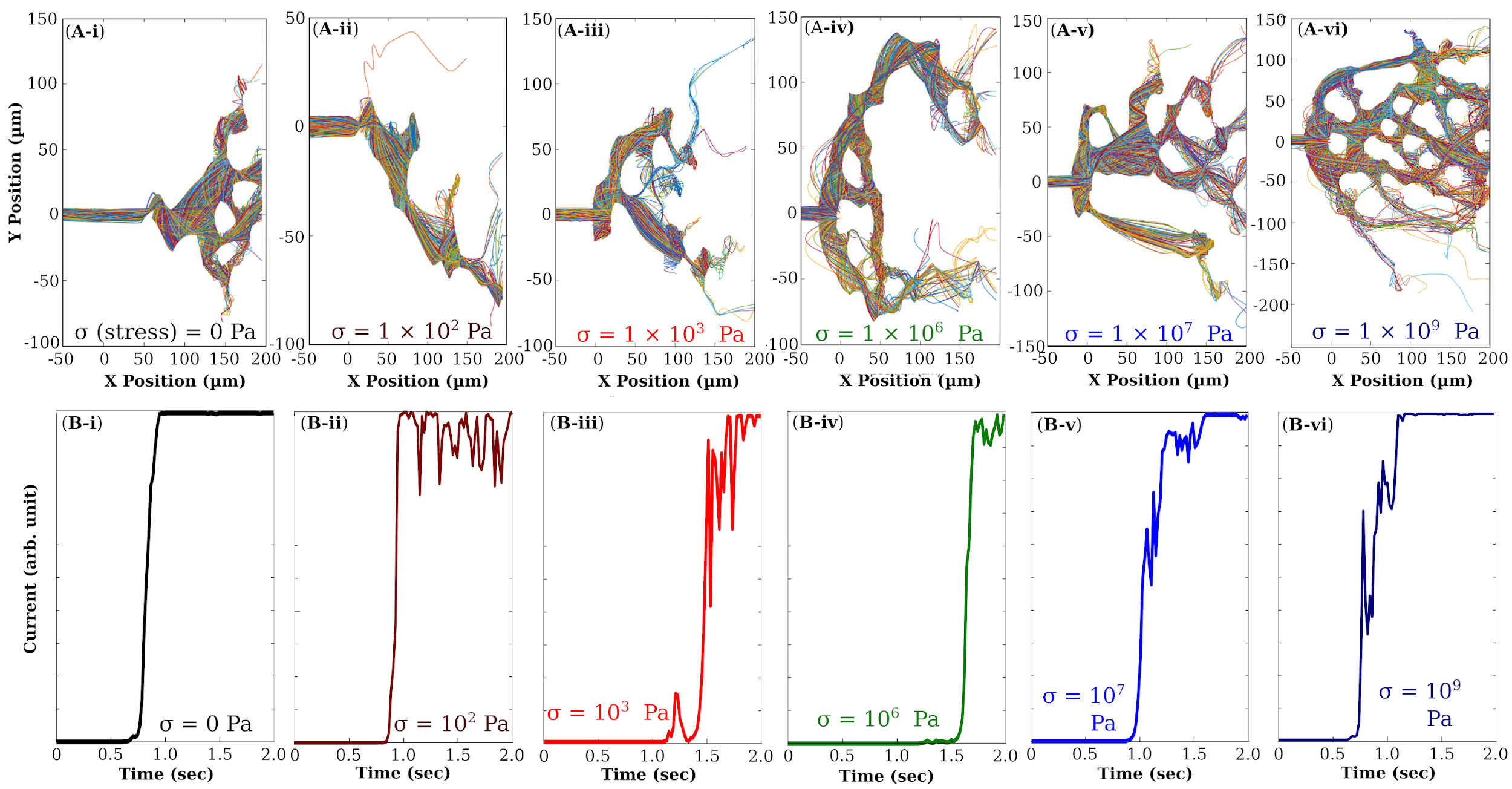


**Figure S10**. Dendrite growth simulation (**A**) and current growth simulation (**B**) under a stress potential from 0 Pa (i) to 1 GPa (ii). The single strand secondary branches densify with increasing stress. There is greater interconnectivity between primary branches and, at the high stress regime, greater interconnectivity between secondary branches. The current growth behaviour shows a starting threshold at latter times until 10^6^ Pa, which then deceases with increasing stress from 10^7^ to 10^9^ Pa. This is likely due to the fast diffusion by silver particles towards the opposing electrode, followed by fast coalescence of all silver particles, all at once, into a dense conducting chain.


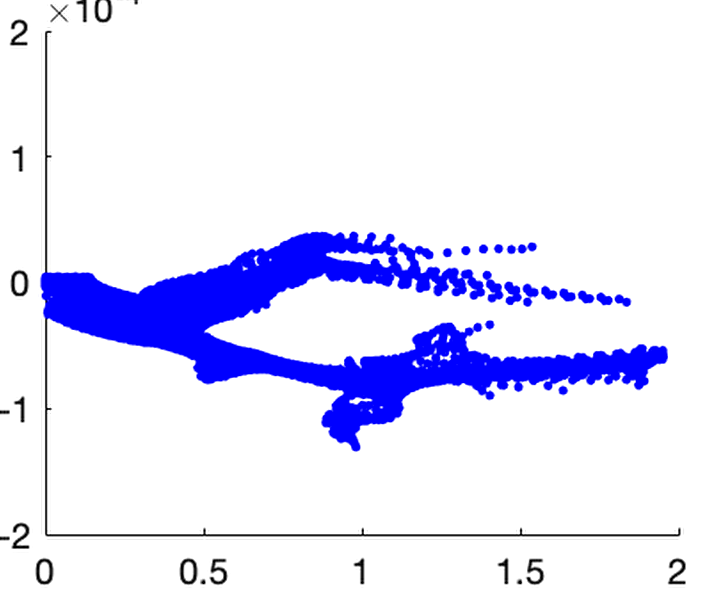

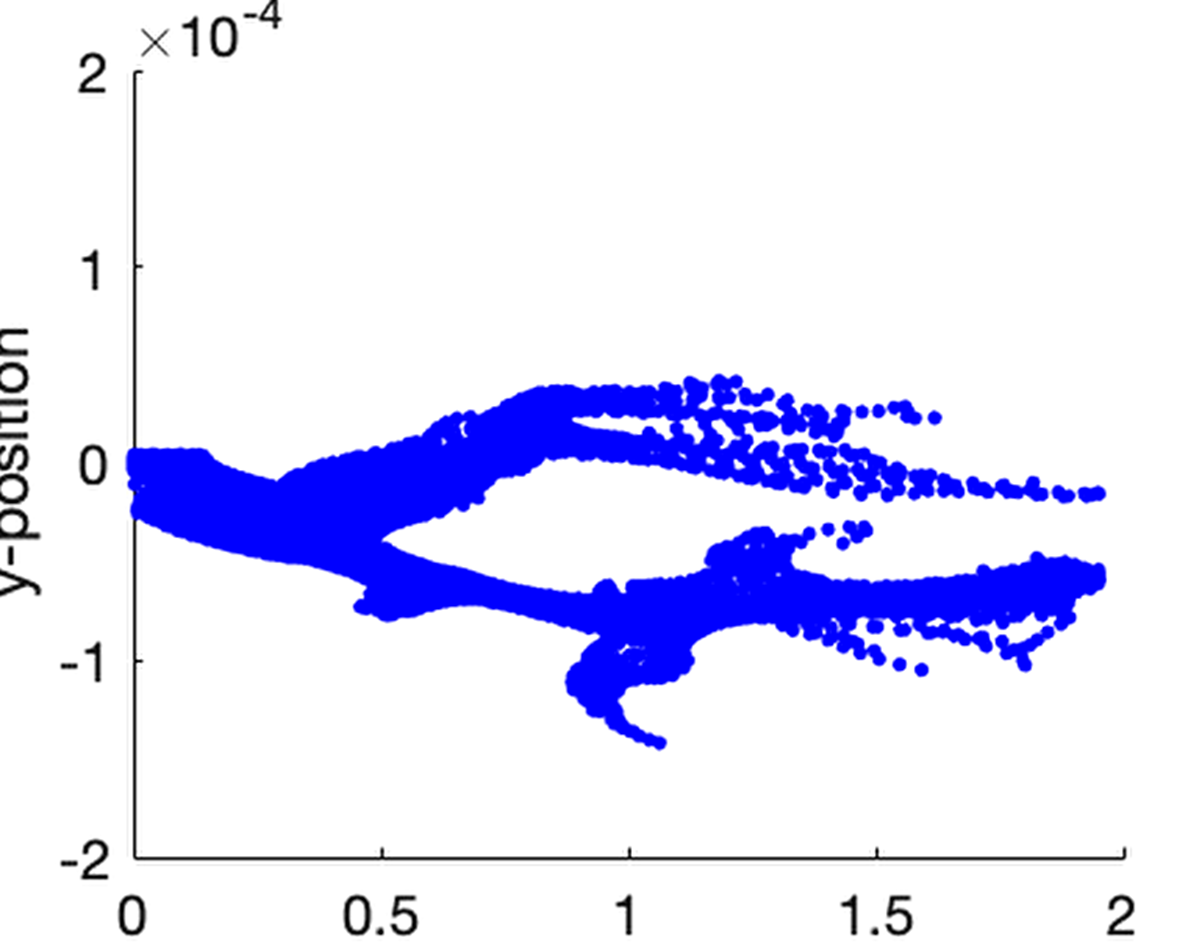

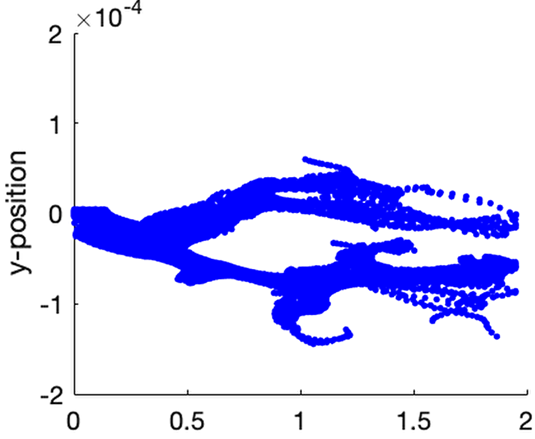


**Figure S10B**. Snapshots of the silver filament growth process simulation under applied electric field and additional intrinsic stress potential. The leading edge particles initially have noticeable gaps between particle. As the filament grows, additional particles fill the gaps and local looping of particles (indicating coalescence) cause the branches to thicken.

**Dendrite Growth Simulations with Compliance Currents**


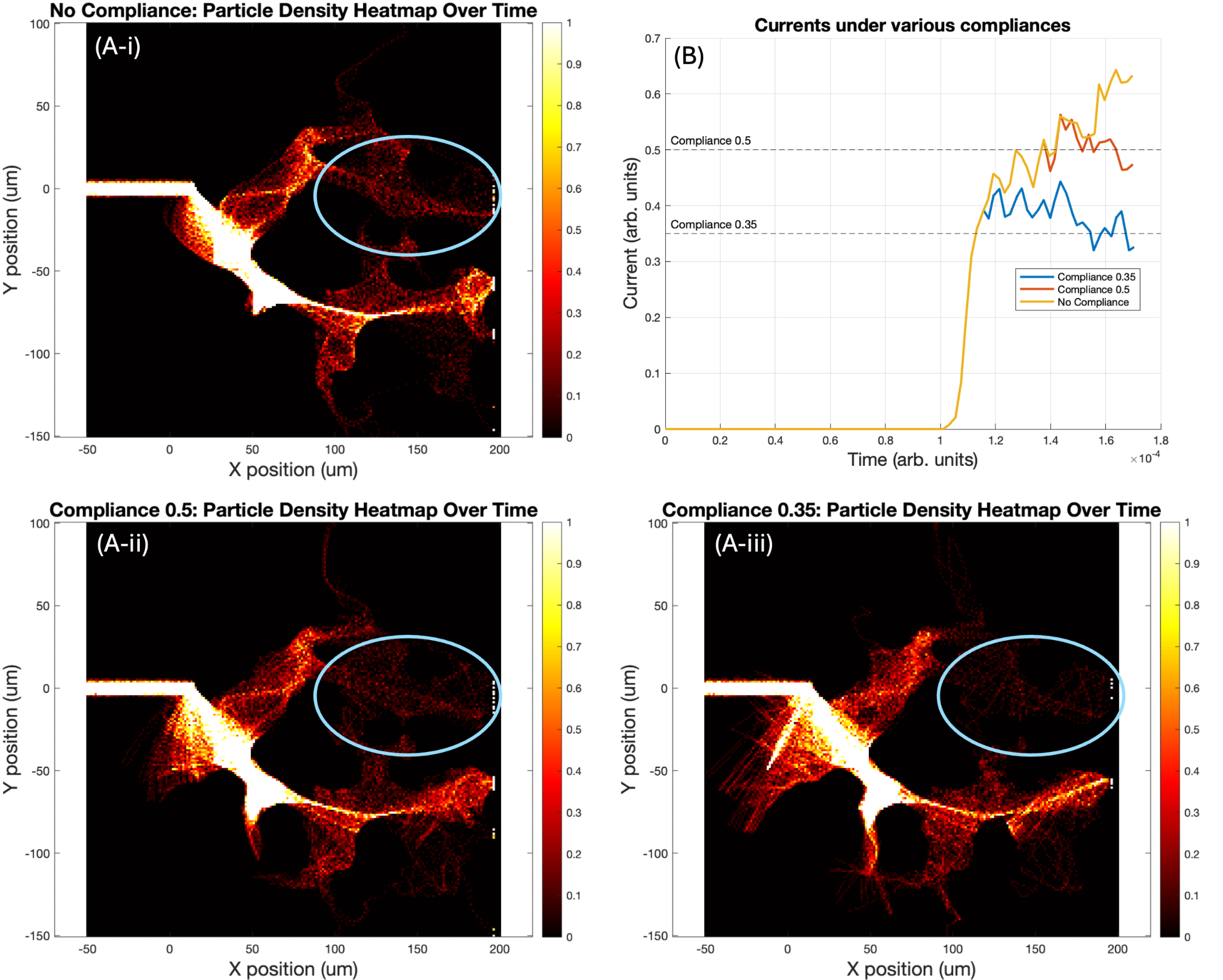


**Figure S11**. Cumulative Ag concentration over time in dendrite growth simulations under varying compliance current levels. **(A-i)** shows a strong secondary branch forming under no compliance limit. **(A-ii)** and **(A-iii)** show comparatively weaker filamentation in secondary branches under compliance limits of 0.5 and 0.35, respectively. The blue circles highlight areas where branching is noticeably diminished. This reduction in secondary growth is attributed to the drop in electric field strength after the compliance limit is reached, which suppresses further dendritic propagation. **(B)** shows the simulated current over time for each condition, highlighting how the applied compliance limit influences filament formation and current saturation.

**Rupturing Process simulated in LAMMPS**

**Figure S12.** LAMMPS simulation of conductive filament rupture following removal of the applied electric field. When the field is turned off, a Langevin thermostat is applied to mimic the effect of Joule heating, which drives rapid diffusion of Ag atoms, causing them to migrate and accumulate in higher-density regions. This redistribution leads to the disconnection of the conductive filament. **(A-i)** and **(A-ii)** show simulation snapshots before and after rupture, revealing the structural changes in the Ag filament. **(B-i)** and **(B-ii)** show corresponding histograms of Ag atom counts along the z-axis, illustrating how atoms from the thinnest part of the filament migrate toward either side, breaking the conductive path.
